# The statistical geometry of material loops in turbulence

**DOI:** 10.1038/s41467-022-29422-1

**Published:** 2022-04-19

**Authors:** Lukas Bentkamp, Theodore D. Drivas, Cristian C. Lalescu, Michael Wilczek

**Affiliations:** 1 Max Planck Institute for Dynamics and Self-Organization, Am Faßberg 17, 37077 Göttingen, Germany; 2 Theoretical Physics I, University of Bayreuth, Universitätsstraße 30, 95447 Bayreuth, Germany; 3 Mathematics Department, Stony Brook University, 100 Nicolls Rd., Stony Brook, NY 11794 USA; 4 School of Mathematics, Institute for Advanced Study, 1 Einstein Dr., Princeton, NJ 08540 USA; 5 Max Planck Computing and Data Facility, Gießenbachstraße 2, 85748 Garching b. München, Germany

**Keywords:** Fluid dynamics, Statistical physics, thermodynamics and nonlinear dynamics

## Abstract

Material elements – which are lines, surfaces, or volumes behaving as passive, non-diffusive markers – provide an inherently geometric window into the intricate dynamics of chaotic flows. Their stretching and folding dynamics has immediate implications for mixing in the oceans or the atmosphere, as well as the emergence of self-sustained dynamos in astrophysical settings. Here, we uncover robust statistical properties of an ensemble of material loops in a turbulent environment. Our approach combines high-resolution direct numerical simulations of Navier-Stokes turbulence, stochastic models, and dynamical systems techniques to reveal predictable, universal features of these complex objects. We show that the loop curvature statistics become stationary through a dynamical formation process of high-curvature folds, leading to distributions with power-law tails whose exponents are determined by the large-deviations statistics of finite-time Lyapunov exponents of the flow. This prediction applies to advected material lines in a broad range of chaotic flows. To complement this dynamical picture, we confirm our theory in the analytically tractable Kraichnan model with an exact Fokker-Planck approach.

## Abstract (web summary)

Turbulent flows are observed in atmosphere, ocean, and technology, with turbulent mixing due to stretching and folding of material elements. The authors analyze a geometric perspective of this process and uncover statistical properties of an ensemble of material loops in a turbulent environment.

## Introduction

**Fig. 1:**
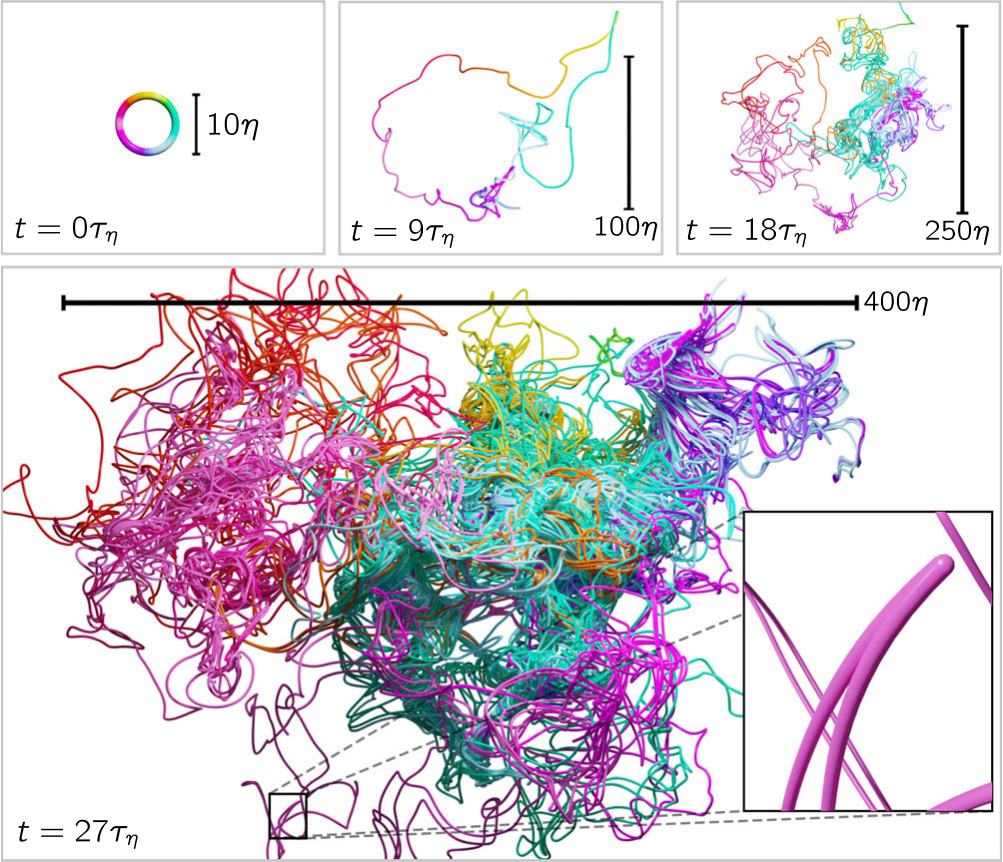
Visualization of material loop evolution. The initially circular loop (color corresponds to initial angle) is advected by a turbulent flow field for 27*τ*_*η*_, where *τ*_*η*_ is the Kolmogorov time. The twisting and folding action of the turbulent flow creates a complex loop geometry while the length of the loop increases exponentially on average (cf. Fig. 3). The loop shown is a comparably extreme case; loops in less turbulent regions develop an extended and complex structure after a longer time. Inset: material fold causing a peak of curvature. (See also Supplementary Movie).

Chaotic flows tend to fold, writhe, and wrinkle material elements into a state of seemingly infinite complexity over time (see Fig. 1 and Supplementary Movie). A fundamental question is whether this tumultuous process has any predictable features which persist over long periods of time. Answering this question provides insights into the process of mixing which occurs in a whole range of systems, from the diffusion of dye into water, the dispersion of plankton colonies on the ocean surface, to the blast propagation in supernovae thermonuclear explosions^1^. Material lines and interfaces, in particular, provide idealized descriptions of nutrient, temperature, and salinity fronts in the oceans^2^, and potential vorticity fronts in the atmosphere^3^. They are also closely related to the dynamics of vorticity filaments in fully developed turbulence^4,5^, the conformation of polymer chains^6–8^, the dynamics of flexible phytoplankton chains^9^, as well as the motion of magnetic field lines at high conductivity (or high magnetic Reynolds numbers)^10^. The latter is related to the dynamo problem, in which chaotic stretching, folding, and twisting processes are essential for sustaining the growth of a magnetic field. The progress we make in understanding how material elements react to turbulent flows stands to advance our understanding of these fundamental problems.

**Fig. 2:**
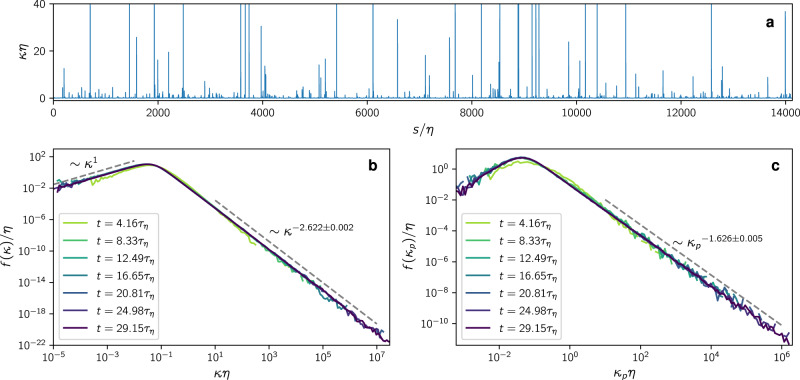
Localized peaks of curvature along the loop cause heavy-tailed curvature distributions. **a** Curvature along a material loop at *t* = 29.15*τ*_*η*_ as a function of arc length *s*. The function is highly spiked, indicating that high curvature only occurs in isolated narrow regions. These isolated peaks contribute to the high-curvature tails of the curvature PDF. **b** Curvature PDF of material loops at times *t* = 4.16*τ*_*η*_ (light green) up to *t* = 29.15*τ*_*η*_ (violet). **c** PDF of curvature peaks of material loops at the same times. The high-curvature regime is fitted by power laws in the regions indicated by the dashed lines by means of a linear fit to the logarithm of the PDF using binomial error estimates.

The geometry of material objects advected and deformed by a turbulent flow can be very complex. While volumes are preserved by incompressible flows, the length of lines and the area of surfaces typically grow exponentially^11–15^, with their geometry appearing fractal^16–18^. Since any curve in space is uniquely described by its curvature and torsion^19^, there have been numerous works attempting to characterize the curvature of material lines but also of material surfaces^20–34^ and Lagrangian trajectories^35–37^. Although material lines seem to become unfathomably complicated over time, the above works suggest that curvature distributions do in fact settle down to a well-defined stationary state which features robust power-law tails (see Fig. 2), sparking hope that certain features can be predicted by theory.

Here we present a line of arguments based on the dynamical mechanism of sling or fold (i.e., curvature peak) formation and its relation to finite-time Lyapunov exponents that leads to a quantitative prediction of the power law of the curvature distribution observed in Fig. 2, panels b and c. We show that the high-curvature regime of the material line can be understood as an ensemble of persistent parabolic folds, which are formed by random stretching of the line. In this way, we illustrate how understanding dynamical mechanisms can be used to make deductions about statistical geometry. For example, our predicted curvature PDF power-law exponent −2.54 ± 0.11 (3% relative error to the measured exponent) implies that, in the long-time limit, the average curvature along advected loops is finite but all higher moments diverge. The only input of our theory is the distribution of Lyapunov exponents of the underlying flow field and, as such, our results apply to a wide range of chaotic dynamics. Our predictions are confirmed by direct numerical simulations of fully developed homogeneous, isotropic Navier-Stokes turbulence as well as by exact results in the analytically solvable Kraichnan model.

## Results

To investigate the evolution of material loops **L**(*ϕ*, *t*) in fully developed turbulence, we consider initially circular loops and parameterize them by the initial angle *ϕ* ∈ [0, 2*π*). Each point of the loop follows the velocity field **u**(**x**, *t*) according to the tracer equation

1 $${\partial }_{t}{{{{{{{\bf{L}}}}}}}}(\phi ,t)={{{{{{{\bf{u}}}}}}}}({{{{{{{\bf{L}}}}}}}}(\phi ,t),t).$$

The evolution of such a loop is shown in Fig. 1, which illustrates that the loop rapidly grows in length and diameter, while attaining a complex geometry due to the stretching and folding by the underlying turbulent flow.

As a key metric to characterize the geometry of the loop, we here focus on the curvature

2 $$\tilde{\kappa }(\phi ,t)=\frac{\left|({\partial }_{\phi }^{2}{{{{{{{\bf{L}}}}}}}})\times ({\partial }_{\phi }{{{{{{{\bf{L}}}}}}}})\right|}{{\left|{\partial }_{\phi }{{{{{{{\bf{L}}}}}}}}\right|}^{3}}.$$

Material lines grow non-uniformly in length over time. Hence for an evolving ensemble of loops, the distribution of curvature can be defined in different ways, depending on the probability measure we associate with the points along the loop. A simple way of defining the probability density function (PDF) of curvature *f*(*κ*; *t*), which does not depend on the initial parameterization, is to take curvature samples uniformly along the arc length of the loops. Specifically,

3 $$f(\kappa ;t)=\frac{1}{\langle L(t)\rangle }\left\langle \int\nolimits_{0}^{L(t)}{{{{{{{\rm{d}}}}}}}}s\,\delta (\kappa -\tilde{\kappa }(s,t))\right\rangle$$

where *δ* is the Dirac delta function, *L*(*t*) is the length of the loop at time *t*, and $\tilde{\kappa }(s,t)$ is the curvature of the loop as a function of arc length *s* at time *t*. The average 〈·〉 is taken to be uniform over loops, and we have here used $\tilde{\kappa }$ to distinguish the loop (realization) dependent curvature from its sample-space variable *κ*.

We use fully resolved turbulence simulations to investigate this measure of the statistical geometry of material lines (see Methods). Here, we focus on a data set at the Taylor-scale Reynolds number *R*_*λ*_ ≈ 216, in which we track 1000 randomly placed loops with an initial diameter of 10*η* (*η* is the Kolmogorov length scale). We test the robustness of our results with additional simulations at various Reynolds numbers in Supplementary Note 1.

The resulting curvature PDF at different times is shown in Fig. 2b. Remarkably, persistent power-law tails form within a few Kolmogorov time scales *τ*_*η*_, which eventually range over several decades of curvature after the loops have been deformed for 29*τ*_*η*_ (~1.5 integral times). Within this observation window, the shape of the distribution appears to become stationary, whereas the support, i.e., the range from minimum to maximum curvature, grows indefinitely in extent. Hence the largest curvatures correspond to structures significantly smaller than the Kolmogorov length scale *η*. As we show in Supplementary Note 1, the distributions are almost indistinguishable for different Reynolds numbers when nondimensionalized by *η*, but they shift to larger *κ* when displayed in units of the integral length. This is a first indication that the curvature distribution is generated by the smallest scales of the flow, in particular by velocity gradients. Given the markedly complex shape of the deformed material loop, the universal shape of the distribution calls for a theoretical explanation, which we develop in the following.

### Ensemble of material folds

The high-curvature regime of the curvature distribution is heavy-tailed and characterized by rare events. Over time, the material line will form isolated sites of extremely high curvature^30–34^, as can be seen in Fig. 2a. Such curvature peaks mark sharp folds in the material line geometry. In the following, we reveal how such folds form stochastically and how this is related to the power-law exponent of the curvature distribution.

This picture in view, we estimate the high-curvature tail of the PDF (3) in the statistically steady state by replacing the ensemble average over entire loops in (3) by an ensemble of folds,

4 $$\displaystyle f(\kappa ) \sim \int\nolimits_{0}^{\infty }{{{{{{{\rm{d}}}}}}}}{\kappa }_{p}\,f({\kappa }_{p})\int\nolimits_{-\infty }^{\infty }{{{{{{{\rm{d}}}}}}}}s\,\delta \left(\kappa -{\kappa }^{{{{{{{\rm{pb}}}}}}}}(s;{\kappa }_{p})\right).$$

Here, *κ*_*p*_ is the peak curvature of a fold and *f*(*κ*_*p*_) its distribution. The second integral is the contribution of curvature around each curvature peak. As we will elaborate in more detail below, high-curvature folds develop a universal, locally parabolic shape. The curvature function around a peak with maximum *κ*_*p*_, therefore, can be estimated as^32^

5 $${\kappa }^{{{{{{{\rm{pb}}}}}}}}(s;{\kappa }_{p})=\frac{{\kappa }_{p}}{{\left(1+{F}^{-1}{(| {\kappa }_{p}s| )}^{2}\right)}^{3/2}},$$

where *F*^−1^(*x*) denotes the inverse of the primitive of $\sqrt{1+{x}^{2}}$ on the positive real line, originating from parameterizing the parabola by arc length. Remarkably, the curvature profile is characterized by the peak curvature as the only parameter. To further evaluate (4), we substitute the inner integration variable by $\kappa ^{\prime} ={\kappa }^{{{{{{{\rm{pb}}}}}}}}(s;{\kappa }_{p})$ with the Jacobian

6 $$\left|\frac{{{{{{{{\rm{d}}}}}}}}{s}^{{{{{{{\rm{pb}}}}}}}}(\kappa ^{\prime} ;{\kappa }_{p})}{{{{{{{{\rm{d}}}}}}}}\kappa ^{\prime} }\right|=\frac{1}{3\kappa {^{\prime} }^{2}\sqrt{{({\kappa }_{p}/\kappa ^{\prime} )}^{2/3}-1}},$$

which yields

7 $$f(\kappa ) \sim \int\nolimits_{\kappa }^{\infty }{{{{{{{\rm{d}}}}}}}}{\kappa }_{p}\,f({\kappa }_{p})\left|\frac{{{{{{{{\rm{d}}}}}}}}{s}^{{{{{{{\rm{pb}}}}}}}}(\kappa ;{\kappa }_{p})}{{{{{{{{\rm{d}}}}}}}}\kappa }\right|.$$

This equation expresses the curvature PDF as a composition of the curvature peak PDF with the contribution from the locally parabolic folds.

### Statistical evolution of curvature peaks

In what follows, we determine the curvature peak distribution *f*(*κ*_*p*_), which can be achieved by capturing the essence of the curvature peak dynamics. Since peaks are generally generated at medium curvature and then grow stochastically, we may define the generation time *t*_0_ of a large peak as the time where it has first surpassed an (arbitrary) threshold *κ*_0_ and its age as *τ* = *t* − *t*_0_. At time *t*, the ensemble of peaks larger than *κ*_0_ can thus be attributed a distribution of ages *f*(*τ*; *t*). By the law of total probability, the peak distribution above *κ*_0_ can be estimated as

8 $$f({\kappa }_{p};t) \sim \int\nolimits_{0}^{t}{{{{{{{\rm{d}}}}}}}}\tau \,f({\kappa }_{p}| \tau )f(\tau ;t),$$

where *f*(*κ*_*p*_∣*τ*) is the probability of a peak with curvature *κ*_0_ at time *t*_0_ to have curvature *κ*_*p*_ at time *t*_0_ + *τ*. This decomposes the curvature peak distribution into a distribution of peaks with a given age and the distribution of ages. In (7), we are interested in the stationary regime $f({\kappa }_{p}):= \mathop{\lim }\nolimits_{t\to \infty }f({\kappa }_{p};t)$, which we expect to be well captured by the estimate (8) and to be independent of the arbitrary threshold *κ*_0_.

**Fig. 3:**
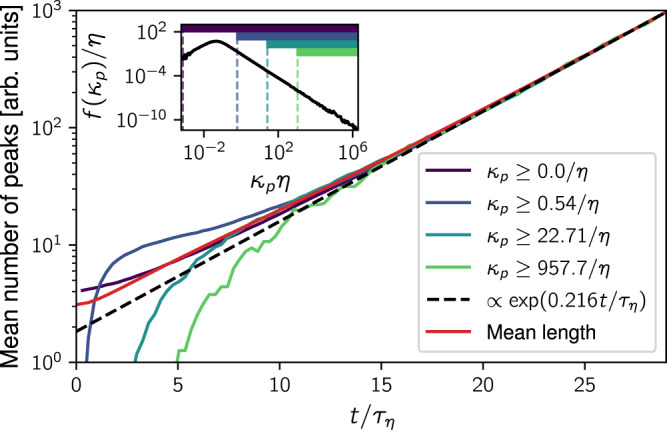
Mean number of curvature peaks above different thresholds over time. The lines are vertically shifted for comparison, showing that the peaks are generated at a clearly defined exponential rate. Moreover, the curves appear to be asymptotically proportional to the mean arc length of loops (red). The dashed line indicates an exponential fit to the last third of the total peak number curve (violet), yielding the rate *β* = (0.21619 ± 0.00014)/*τ*_*η*_. The standard error of this rate is so small that we neglect it in the following. Note that without vertically shifting the curves in the plot, they would remain ordered as a function of the threshold condition. Inset: Curvature peak distribution at *t* = 29.15*τ*_*η*_ indicating the different thresholds.

The peak age distribution can be estimated from the mean number of curvature peaks. Figure 3 shows that the mean numbers of curvature maxima above different thresholds grow at the same exponential rate *β* ≈ 0.216/*τ*_*η*_, which coincides with the growth rate of the mean length of the loops. Intuitively, this can be explained by the fact that the generation of folds is a random process along the loop. Since the loop length grows on average exponentially over time, so does the number of folds. Neglecting the disappearance of peaks, we, therefore, estimate the probability of a high-curvature fold at time *t* to be generated before some time $t^{\prime}$ (with $0\le t^{\prime} \le t$) by the fraction of peaks that existed at $t^{\prime}$, given by ${e}^{\beta t^{\prime} }/{e}^{\beta t}$. This cumulative distribution function of peak birth times implies the probability density function of peak age

9 $$f(\tau ;t)\approx \beta {e}^{-\beta \tau },\qquad 0\le \tau \le t.$$

This shows that, since curvature peaks are generated at an exponential rate, their age distribution also decays exponentially, implying that the bulk of the peaks is young even after a long evolution of the loop.

In the following, we investigate the dynamics and statistics of peak curvature in an effort to estimate the remaining conditional probability *f*(*κ*_*p*_∣*τ*) and form our theory.

### Amplification of folds by turbulent stretching

**Fig. 4:**
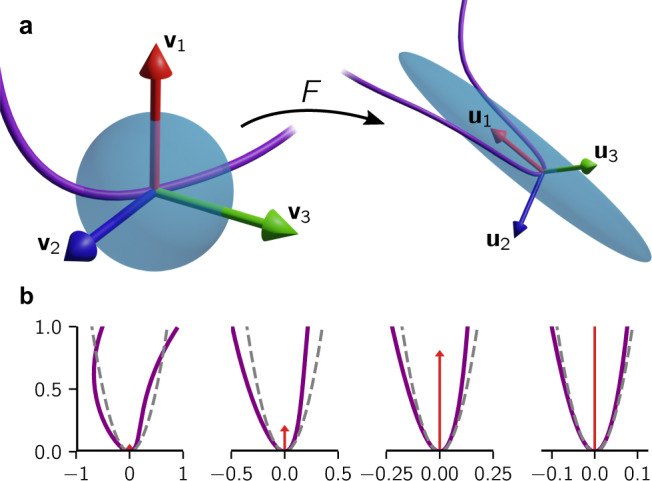
Formation of a parabolic fold. **a** Illustration of the deformation tensor *F*. **v**_*i*_ denote the principal axes of stretching before deformation and **u**_*i*_ the corresponding axes after deformation. A fluid element (blue) will be predominantly stretched along the direction of most stretching **v**_1_ and compressed in the direction of most compression **v**_3_ over time. If a material line element (violet) is initially orthogonal to the direction of most stretching, a fold will form. Such a fold is then compressed onto the **u**_1_–**u**_2_ plane and tends to align with the **u**_1_ direction along which it is amplified. **b** Stretching creates a locally parabolic curve. An initially non-parabolic curve is stretched vertically as indicated by the red arrows. Viewed on the appropriate horizontal scale, the line becomes increasingly parabolic. For comparison, the dashed line indicates a parabola with the same peak curvature.

We observe that those rare peaks that have existed for a long time can exhibit extremely high curvature. This is caused by fluid element stretching, a process quantitatively captured by the deformation tensor

10 $${F}_{ij}({{{{{{{\bf{x}}}}}}}},t)=\frac{\partial {X}_{i}({{{{{{{\bf{x}}}}}}}},t)}{\partial {x}_{j}},$$

where **X**(**x**, *t*) is the Lagrangian map, mapping the initial condition **x** of a tracer particle to its position **X** at time *t*. The singular value decomposition of the deformation tensor associates two coordinate systems **v**_*i*_ and **u**_*i*_ with the deformation (see Methods), as illustrated in Fig. 4a. The associated exponential stretching rates are given by the finite-time Lyapunov exponents (FTLE) *ρ*_*i*_(*t*).

As discussed in ref. ^32^, generically a line element will align with the **u**_1_-direction and become stretched exponentially with ${e}^{{\rho }_{1}(t)t}$ (whose mean asymptotically scales like *e*^*βt*^). The surrounding curve will be forced into the **u**_1_–**u**_2_ plane by compression in the **u**_3_-direction. The dominant stretching in the **u**_1_-direction locally decreases curvature. However, an exception to this generic setting occurs at a finite number of points along the loop when the initial material line lies perpendicular to **v**_1_ (see Fig. 4). In this case, the line element cannot align with **u**_1_ and will align with **u**_2_ instead. The surrounding curve, however, still experiences the stretching in the **u**_1_-direction. This essentially magnifies the local structure of the curve, which will generically result in a parabolic shape, as illustrated in Fig. 4b. Therefore parabolas become increasingly good local approximations of the folds.

To reveal the role of the finite-time Lyapunov exponents, let us consider a parabola *y* = *κ*_0_*x*^2^/2 which is already initially lying in the **v**_1_–**v**_2_ plane. Over time, it is subject to stretching $y^{\prime} ={e}^{{\rho }_{1}(t)t}y$ and $x^{\prime} ={e}^{{\rho }_{2}(t)t}x$, which preserves the parabolic shape, i.e., $y^{\prime} ={e}^{[{\rho }_{1}(t)-2{\rho }_{2}(t)]t}{\kappa }_{0}x{^{\prime} }^{2}/2$. In this process, the peak curvature increases as long as *ρ*_1_(*t*) > 2*ρ*_2_(*t*)^32^, i.e., the first FTLE must be more than twice as large as the second one. We illustrate this at the example of a parabola in a linearized flow in Methods, showing that its peak curvature grows as

11 $${\kappa }_{p}(t)\mathop{\approx }\limits^{t\gg 0}{\widetilde{\kappa }}_{0}{e}^{[{\rho }_{1}(t)-2{\rho }_{2}(t)]t}$$

for some effective initial peak curvature ${\widetilde{\kappa }}_{0}$. This equation can already be found in ref. ^32^, where it is derived for a generic material line. Let us call the growth rate of peaks *ρ*_*p*_(*t*) = *ρ*_1_(*t*) − 2*ρ*_2_(*t*). In turbulence, this growth rate is typically asymptotically positive. In our simulation used for obtaining the FTLEs (see Methods), we can estimate the infinite-time Lyapunov exponents, ${\lambda }_{i}=\mathop{\lim }\nolimits_{t\to \infty }{\rho }_{i}(t)$, by taking the mean of the FTLEs at the final time of the simulation, which yields *λ*_1_ ≈ 0.12/*τ*_*η*_, *λ*_2_ ≈ 0.03/*τ*_*η*_, *λ*_3_ ≈ −0.15/*τ*_*η*_, in good agreement with previous literature^38,39^, and thus ${\lambda }_{p}=\mathop{\lim }\nolimits_{t\to \infty }{\rho }_{p}(t)\approx 0.06/{\tau }_{\eta } \; > \; 0$.

### Connecting the power-law exponent to fluid stretching

To relate the dynamical formation of folds to the power-law tails of the curvature PDF, we estimate the distribution of *κ*_*p*_(*t*) by making statements about the distribution of FTLEs. By ergodicity, FTLEs behave like sums of independent and identically distributed random variables at large times^39,40^. The same is true for the growth rate of peaks *ρ*_*p*_(*t*). Using its Cramér function *S*(*ρ*_*p*_), we make a large-deviations estimate of the PDF,

12 $$f({\rho }_{p};t)\approx N(t){e}^{-tS({\rho }_{p})},$$

where *N*(*t*) is a normalization. Transforming by (11), the peak curvature PDF for peaks of age τ can thus be written as

13 $$f({\kappa }_{p}| \tau )\approx \frac{N(\tau )}{{\kappa }_{p}\tau }{e}^{-\tau S\left(\log \left(\frac{{\kappa }_{p}}{{\kappa }_{0}}\right)/\tau \right)}.$$

Note that we here identified the peak age *τ* with the time *t* and the curvature threshold *κ*_0_ with the effective initial peak curvature ${\widetilde{\kappa }}_{0}$. For the asymptotics that we are interested in, the distinction does not matter. Inserting this result into (8), combined with (9) and letting *t* → *∞*, gives the asymptotic distribution of curvature peaks in the high-curvature regime

14 $$f({\kappa }_{p}) \sim \int\nolimits_{0}^{\infty }{{{{{{{\rm{d}}}}}}}}\tau \,{e}^{-\beta \tau }\frac{N(\tau )}{{\kappa }_{p}\tau }{e}^{-\tau S\left(\log \left(\frac{{\kappa }_{p}}{{\kappa }_{0}}\right)/\tau \right)}.$$

We now use the method of steepest descent^41^ in order to extract the large-*κ*_*p*_ asymptotics of the peak curvature distribution from our estimate (14). The result (see Methods) is that the distribution scales as a power law, $f({\kappa }_{p}) \sim {\kappa }_{p}^{-1-\alpha }$, with exponent

15 $$\alpha =\mathop{\min }\limits_{{\rho }_{p}}\left[\frac{1}{{\rho }_{p}}(\beta +S({\rho }_{p}))\right].$$

**Fig. 5:**
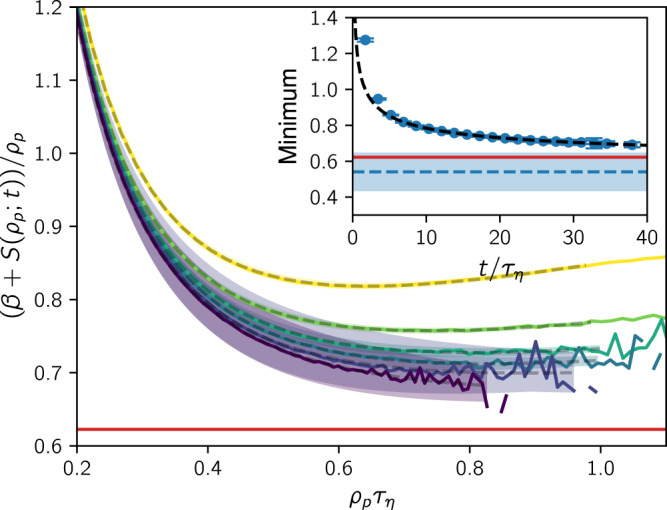
Determination of the steepest-descent minimum. The Cramér function is estimated from FTLE histograms by (12). We call these finite-time estimates *S*(*ρ*_*p*_; *t*). Here we show the function minimized in (15) for estimates of the Cramér function ranging from *t* = 6.90*τ*_*η*_ (yellow) up to *t* = 39.68*τ*_*η*_ (violet). Best fits are indicated by dashed lines with shaded areas showing their error (see Methods for details). Inset: Minima of these functions over time. A simple fit of the decay of minima (black dashed line) yields an estimate of their limiting value *α* = 0.54 ± 0.11 (horizontal blue dashed line and shaded area). For comparison, the red lines show the value of *α* estimated by subtracting 2 from the observed curvature PDF power-law exponent in Fig. 2b, showing a good agreement within uncertainties. For more details, see Methods.

This minimum is estimated for our data in Fig. 5, where the Cramér functions have been estimated via (12) using FTLE histograms from an additional simulation (see Methods). While we are interested in finding the minimum for the fully converged Cramér function, the amount of samples needed to resolve large-deviations statistics increases exponentially with time, limiting our observation window of the minimum to a maximum time of about 30 to 40*τ*_*η*_. In this regime, the minima still lie above the value of *α* inferred from the loops simulation (red line). However, an analysis of the time evolution of minima (Fig. 5, inset) reveals that they are well described by a slow, algebraic decay. Extrapolating the desired minimum toward *t* → *∞*, we get the estimate *α* = 0.54 ± 0.11, slightly below but within error bars of the curvature peak power-law exponent in Fig. 2c. For more details on the extrapolation, see Methods.

Given the power-law scaling of the peak distribution, $f({\kappa }_{p}) \sim {{\kappa }_{p}}^{-1-\alpha }$, we can perform the integral (7) to obtain the prediction for the curvature PDF

16 $$f(\kappa ) \sim {\kappa }^{-2-\alpha }.$$

Hence the difference between the curvature power-law exponent and the curvature peak power-law exponent is 1. This difference originates from the curvature contributions of parabolic fold profiles around the peak curvature (5). Comparing Fig. 2b and c shows that this result is consistent with the fully resolved loops simulations. Likewise, our prediction based on Lyapunov exponents estimated by extrapolating the minimum in Fig. 5 captures the observed power-law exponents of both the curvature and curvature peak PDFs very well. In Supplementary Note 1, we explore our result at various Reynolds numbers, with comparable or even better agreement depending on how far the minima can be resolved in time. Therefore, as a central result, we can quantitatively relate the statistical geometry as characterized by the curvature PDF to the formation of folds and the statistics of FTLEs that determine their dynamical evolution.

Interestingly, an alternative formulation of our result can be obtained by using the Legendre transform of the Cramér function, which is known as the generalized Lyapunov exponent^39^. It can be shown (see Methods) that *α* is given implicitly by

17 $$\left\langle {e}^{\alpha {\rho }_{p}(t)t}\right\rangle \sim \left\langle {e}^{{\rho }_{1}(t)t}\right\rangle$$

in the large-deviations approximation, where ~ indicates the same exponential scaling for large *t*. This can be understood as the statement that the power-law exponent is chosen so that curvature peak generation (represented by the line growth rate *ρ*_1_(*t*)) and peak amplification (represented by the peak curvature growth rate *ρ*_*p*_(*t*) = *ρ*_1_(*t*) − 2*ρ*_2_(*t*)) are on average balanced. For example, in a flow with the same peak amplification (same statistics of *ρ*_*p*_(*t*)) but stronger line growth (larger $\langle {e}^{{\rho }_{1}(t)t}\rangle$) and thus stronger peak generation, a larger fraction of small-curvature peaks will accumulate until the stationary state is reached. This means that the curvature PDF in the stationary state has to decay faster, corresponding to a larger *α*, as encoded in (17). We explore this result numerically in Supplementary Note 2, showing that this complementary way of computing *α* comes equally close to the value observed in the loops simulations.

### Exact results in the Kraichnan model

To demonstrate the robustness of our results beyond Navier-Stokes turbulence, we consider the exactly solvable Kraichnan model^42^. The Kraichnan model of turbulence replaces the advecting velocity with a spatially correlated Gaussian random field, white in time, which mimics turbulence. While we do not expect the predictions of the curvature PDF power law from the Kraichnan model to be in quantitative agreement with our DNS results, it serves as a test case in which our approach can be compared rigorously against exact independent Fokker-Planck calculations.

In this setting, all of our argumentation about fold formation and its statistical implications can be made exact. First, the Cramér function takes the parabolic form^40^

18 $$S({\rho }_{p})=\frac{{({\rho }_{p}-{\lambda }_{p})}^{2}}{2{D}_{p}},$$

with *λ*_*p*_ = 3*Q*, *D*_*p*_ = 14*Q* and *Q* a constant related to the energy spectrum quantifying fluctuations of the velocity gradient (see Methods). *λ*_*p*_ and *D*_*p*_/*t* are the mean and variance of the Gaussian distribution of *ρ*_*p*_ that can be computed from the known multivariate Gaussian distribution of the *ρ*_*i*_^40^. Now, the integral (14) can be performed exactly, yielding a power law ${{\kappa }_{p}}^{-1-\alpha }$ with

19 $$\alpha =-\frac{{\lambda }_{p}}{{D}_{p}}+\sqrt{\frac{{\lambda }_{p}^{2}}{{D}_{p}^{2}}+\frac{2\beta }{{D}_{p}}}.$$

The growth rate of the mean length of line elements in the Kraichnan model is *β* = 4*Q*, determined by ${e}^{\beta t} \sim \langle {e}^{{\rho }_{1}(t)t}\rangle$. This evaluates to *α* = 4/7, a curvature peak PDF power law − 11/7, and a curvature PDF power law − 18/7 ≈ −2.571. Although this is very close to the exponent − 2.622 ± 0.002 that we find in Navier-Stokes turbulence, we believe that our measurements are precise enough to conclude that the exponents are in fact different and that their closeness is coincidental.

Importantly, this result based on our picture of curvature growth due to fold formation is consistent with an independent, complementary approach facilitated by the rapidly fluctuating velocity field. Using Itô calculus, one can obtain an exact Fokker-Planck equation for the curvature distribution (see Methods) and study its steady state. The equation takes the form

20 $${\partial}_{t}\, f=-{\partial }_{\kappa }\left(-18Q\kappa f-7Q{\kappa }^{2}{\partial }_{\kappa }\, f+\frac{9P}{\kappa }f-9P{\partial }_{\kappa }\, f\right),$$

and features the stationary solution

21 $$f(\kappa )=\frac{1}{{{{{{{{\mathcal{Z}}}}}}}}}\kappa {\left(9P+7Q{\kappa }^{2}\right)}^{-25/14},$$

where *P* is a constant quantifying fluctuations of second-order derivatives of velocity (see Methods) and ${{{{{{{\mathcal{Z}}}}}}}}$ is the normalization constant. This exact solution transitions between a *κ*^1^ power law in the small-curvature regime and a *κ*^−18/7^ power law in the large-curvature regime. Hence our framework based on the dynamical evolution of curvature peak statistics and Itô calculus yield exactly the same large-curvature exponent. The shape of the PDF is also in qualitative agreement with our numerical observations in Navier-Stokes turbulence, see Fig. 2b. A numerical analysis of the Kraichnan case can be found in Supplementary Note 7. Analogous computations^43^ have been done for the curvature PDF of magnetic field lines in the context of the turbulent dynamo problem without compensating for arc length.

We remark in passing that it would be interesting to study material line curvature statistics in the compressible *d*-dimensional Kraichnan model^43^ also from the complementary perspective of fold formation. There, the compressibility can be parameterized by an index *℘* and Lyapunov exponents can be explicitly computed (see §2.4 of ref. ^44^). The chaotic phase characterized by positive leading Lyapunov exponent *λ*_1_ > 0 occurs when *℘* < *d*/4. In this regime, one can vary *λ*_*p*_ = *λ*_1_ − 2*λ*_2_ and analytically study its effect on curvature statistics. As such, the compressibility can be used to precisely control the curvature statistics.

## Discussion

We investigated the curvature statistics of material loops in fully developed turbulence to characterize their statistical geometry. We find that the curvature PDF rapidly converges to a stationary distribution and establish a theory of curvature peaks forming along the loop to explain the power law in its high-curvature regime. Using the connection between curvature peak dynamics and finite-time Lyapunov exponents, we are able to theoretically link the power-law exponent to FTLE large-deviations statistics. In Navier-Stokes turbulence, we find our theory to be in very good agreement with direct numerical simulations. In the Kraichnan model, our theoretical prediction agrees precisely with exact analytical calculations.

An important issue concerns how the results presented here depend on the Reynolds number. In Supplementary Note 1, we provide numerical evidence that moderate variations of the Reynolds number lead qualitatively to the same picture with only very slight quantitative changes in the power-law exponents. When nondimensionalized by the Kolmogorov length scale, the curvature PDFs for different Reynolds numbers collapse in very good approximation, consistent with the notion that turbulent stretching and folding is driven by the tentatively universal small-scale velocity gradients in turbulence. In light of this, it seems plausible to us that the shape of the curvature distribution we observe is universal and will persist in the limit of large Reynolds number.

Our methods and theoretical predictions can be applied to a large class of chaotic flows and can thereby provide a new statistical-geometry perspective on the intricacies of their evolution. Since a host of processes are closely related to the transport of material lines, our results may help to shed light on such problems from biophysics, geophysics and astrophysics. For example, in polymer turbulence, the conformation tensor describing polymeric stresses is a materially transported quantity modified by (internal) restoration forces. As such, our computational and theoretical techniques used to study ideal material transport in the form of material lines, suitably adapted to accommodate internal degrees of freedom, provide a framework to study fluid–polymer interaction.

Our work may also shed new light on classical questions in magnetohydrodynamic (MHD) turbulence and, in particular, the dynamo problem. For example, curvature PDFs of magnetic field lines in MHD have been observed to form power-law tails in the kinematic stage^43^. It would be very interesting to study how this is related to the formation of folds in the magnetic field and how these folds behave in the non-linear stage of the turbulent dynamo. Furthermore, it is well known that flux cancellations in turbulent magnetic dynamos occur in part due to the folding/bundling of magnetic field lines^45,46^. In fact, our simulations indicate that tightly wound bundles along the loop are in close correspondence with curvature peaks (see Supplementary Note 3). Thus the statistical attributes (generation and growth rates) of the peaks predicted here may be indicative of the genericity and intensity of configurations that can stifle dynamo growth. It is also known that magnetic helicity—a measurement of the linkage, twist, and writhe of magnetic loops—has a profound effect on the growth rates for the dynamo^47^. The tools developed here can be used to study field lines in MHD in the highly conductive regime. Conditioning on the level of magnetic helicity, they could thus offer a new geometric perspective on the role that magnetic helicity plays in dynamo action.

Finally, we remark that it would be of great interest to generalize our framework to accommodate higher-dimensional structures, such as material surfaces. A material surface can be understood as a continuous family of material lines. We therefore expect it to form folds extending as one-dimensional structures across the surface. This could then be applied to study interfacial problems such as the dispersion of algae blooms or oil spills in the ocean, where the description of the boundary’s geometry is of crucial importance for prediction.

## Methods

### Navier-Stokes simulations for loop tracking

For the direct numerical simulations (DNS), we use our code TurTLE^48^. It implements a pseudo-spectral solver for the Navier-Stokes equation in the vorticity formulation with a third-order Runge-Kutta method for time stepping and a high-order Fourier smoothing^49^ to reduce aliasing errors. The flow is forced on the large scales by maintaining a fixed energy injection rate in a discrete band of Fourier modes at small wavenumbers *k* ∈ [1.0, 2.0] (DNS units). The simulations presented here were computed on 1024^3^ grid points with a small-scale resolution *k*_max_ *η* ≈ 2.9, where *k*_max_ is the maximum resolved wavenumber. Using the same initial background flow, we conducted two separate simulations with different sets of Lagrangian tracers.

The first simulation contains 10^3^ initially circular loops of diameter ~10*η* with random position and orientation. Each sample point of the loops is treated as a Lagrangian tracer particle. Over time, the strongly heterogeneous line stretching necessitates an adaptive refinement of the loops^50,51^. Using fifth-order B-spline interpolation^52^, we determine the arc length between adjacent sample points in time intervals of 0.16*τ*_*η*_. Whenever their distance surpasses 0.1*η*, we insert new sample points along the smooth spline curves, which ensures that derivatives of the curves up to fourth order and hence their curvature are well-defined. In order to better resolve high-curvature regions, we additionally require that the distance between sample points does not surpass 1/(6*κ*). This significantly improves the resolution of the large-curvature tail of the curvature PDF. Due to the refinement, the initial total number of sample points across all loops—about 3 × 10^5^—increases to about 1.5 × 10^8^ sample points at 29*τ*_*η*_. The adaptive insertion of particles prohibits the direct use of multi-step methods for particle time stepping. For this simulation, we therefore resort to first-order Euler time stepping of particle trajectories. They are coupled with spline interpolation of the field with continuous derivatives up to and including third order computed over a kernel of 12^3^ grid points (as detailed in ref. ^53^). We verify our determination of the curvature distribution for different temporal and spatial resolutions of the loops in Supplementary Note 4.

While the statistical geometry of any type of material line could be equally well studied, we focus here on material loops due to their important role in fluid dynamics. For example, the velocity circulation along any material loop is invariant in inviscid incompressible fluid motion—a fact known as the Kelvin theorem. While this invariance breaks down in the presence of any non-ideal effect such as viscosity, properties of material loops at high Reynolds number—a regime in which the flow is nearly inviscid – may shed light on a variety of features of fully developed turbulence such as anomalous dissipation and spatio-temporal intermittency^54^. Material loops also arise naturally in the context of astrophysics where they approximately describe the motion of closed field lines of a magnetic field at high magnetic Reynolds numbers in a stellar or planetary system.

### Computation of finite-time Lyapunov exponents and the Cramér function

The second simulation contains 10^8^ uniformly distributed Lagrangian tracers. Along with their trajectories, we integrate the deformation tensor (10). Time stepping is performed using the Heun method coupled with spline interpolation of the field with continuous derivatives up to and including second order computed over a kernel of 8^3^ grid points. In order to ensure numerical stability, we perform a QR-decomposition of the deformation tensor^55^ after each time step and store principal axes and logarithmically scaled stretching factors separately. While in theory the FTLEs are defined by the singular value decomposition, we here use the logarithmic stretching factors obtained from the QR-decomposition as proxies (as done in refs. ^8,38,39^). In certain regimes, their large-deviations statistics may differ^39^. However, in Supplementary Note 8, we show that our theoretical argument can also be made for the proxies. We therefore expect no differing results in the two cases. We then determine finite-time Cramér functions *S*(*ρ*_*p*_; *t*) from the FTLE histograms *f*(*ρ*_*p*_; *t*) as^39^

22 $$S({\rho }_{p};t)=-\log (f({\rho }_{p};t))/t,$$

which converge to the actual Cramér function over time. Given that the Cramér function is known to take its minimum at *S*(*λ*_*p*_) = 0, where ${\lambda }_{p}=\mathop{\lim }\nolimits_{t\to \infty }{\rho }_{p}(t)$, we may accelerate convergence by vertically shifting the finite-time Cramér functions such that their minimum is zero, as done similarly in ref. ^39^. The resulting functions are used as input for Fig. 5.

We determine least-square fits of the finite-time Cramér functions using a Batchelor interpolation between two power laws (corresponding to stretched exponentials for the FTLE PDF),

23 $$S({\lambda }_{p}(t)+x/{\tau }_{\eta };t)=\frac{a{x}^{2}}{{(b+{x}^{2})}^{c}},$$

where *λ*_*p*_(*t*) is the position of the minimum of *S*(*ρ*_*p*_; *t*), and *a*, *b*, and *c* are fitting parameters. In order to obtain fits with reasonable accuracy, we restrict the fitting range to the interval of interest [*λ*_*p*_(*t*), 1/*τ*_*η*_]. If the finite-time Cramér functions take infinite values in this range, then we further restrict the fitting range to their finite values. In order to obtain the error bars in Fig. 5, we vary the fitting parameters within their standard error interval and take the minimum and maximum of the resulting functions. Taking the minimum of the best fits and of their error envelopes, we obtain the time series of minima in the inset of Fig. 5. If a fit takes its minimum at the last value of the fitting range, then this value is omitted.

In order to extrapolate the minimum towards *t* → *∞*, we determine the best fit of the minima time series *m*(*t*) weighted by the errors using an algebraic decay,

24 $$m(t)=A+{\left(\frac{B}{t}\right)}^{C},$$

where *A*, *B*, and *C* are fitting parameters. In order to robustly capture the asymptotic decay using this simple fit function, we leave out an initial transient regime of data points for the fit. We choose $t\ge {t}_{\min }\approx 6.9{\tau }_{\eta }$, where the weighted mean squared error of the fit reaches a plateau, i.e., the point at which the fit improvement from removing more data points diminishes (for more details, see Supplementary Note 1). The parameters are estimated as *A* = 0.54 ± 0.11, *B* = (0.19 ± 0.15)*τ*_*η*_, and *C* = 0.36 ± 0.18.

Note that the overall fitting procedure is very delicate and different choices may lead to different results. The present analysis is our best effort to systematically compute the limiting value of the minima.

### Peak curvature dynamics of a parabola

Here, we determine the evolution of the peak curvature of a fold modeled by a parabola,

25 $${{{{{{{\bf{L}}}}}}}}(\phi ,t)=\; {{{{{{{\bf{L}}}}}}}}({\phi }_{0},t)+(\phi -{\phi }_{0}){{{{{{{\bf{l}}}}}}}}(t){{\Delta }}s +{\kappa }_{p}(0)\frac{{(\phi -{\phi }_{0})}^{2}}{2}{{{{{{{\bf{k}}}}}}}}(t){{\Delta }}{s}^{2},$$

where *ϕ*_0_ is the initial peak position, *κ*_*p*_(0) is its initial peak curvature, **l** and **k** are two initially orthonormal vectors, and Δ*s* is the arc length per angle of the initial parameterization at *ϕ*_0_. In a sufficiently small range of *ϕ* around *ϕ*_0_, the velocity field can be linearized. Then the parabolic shape is preserved and the dynamics of **l** and **k** in the Lagrangian frame is determined by the velocity gradient,

26 $$\frac{{{{{{{{\rm{d}}}}}}}}{{{{{{{\bf{l}}}}}}}}}{{{{{{{{\rm{d}}}}}}}}t} 	={{{{{{{\bf{l}}}}}}}}\cdot \nabla {{{{{{{\bf{u}}}}}}}}({{{{{{{\bf{L}}}}}}}}({\phi }_{0},t),t)\quad \,{{{{{{\rm{and}}}}}}}\,\\ \frac{{{{{{{{\rm{d}}}}}}}}{{{{{{{\bf{k}}}}}}}}}{{{{{{{{\rm{d}}}}}}}}t} 	={{{{{{{\bf{k}}}}}}}}\cdot \nabla {{{{{{{\bf{u}}}}}}}}({{{{{{{\bf{L}}}}}}}}({\phi }_{0},t),t).$$

By (2), the curvature of the fold is given by

27 $$\kappa (\phi ,t)={\kappa }_{p}(0)\frac{\left|{{{{{{{\bf{k}}}}}}}}(t)\times {{{{{{{\bf{l}}}}}}}}(t)\right|}{{\left|{{{{{{{\bf{l}}}}}}}}(t)+{{\Delta }}s(\phi -{\phi }_{0}){\kappa }_{p}(0){{{{{{{\bf{k}}}}}}}}(t)\right|}^{3}}$$

28 $$={\kappa }_{p}(0)\frac{{\left({\left|{{{{{{{\bf{k}}}}}}}}(t)\right|}^{2}{\left|{{{{{{{\bf{l}}}}}}}}(t)\right|}^{2}-{({{{{{{{\bf{k}}}}}}}}(t)\cdot {{{{{{{\bf{l}}}}}}}}(t))}^{2}\right)}^{1/2}}{{\left|{{{{{{{\bf{l}}}}}}}}(t)+{{\Delta }}s(\phi -{\phi }_{0}){\kappa }_{p}(0){{{{{{{\bf{k}}}}}}}}(t)\right|}^{3}}.$$

Over time, **l**(*t*) and **k**(*t*) cease to be orthogonal and the curvature peak position is shifted. Minimizing the denominator yields the new peak position

29 $$\begin{array}{rc}{\phi}_{p}(t)&={\phi}_{0}-\frac{{{{{{{{\bf{k}}}}}}}}(t)\cdot {{{{{{{\bf{l}}}}}}}}(t)}{{{\Delta }}s{\left|{{{{{{{\bf{k}}}}}}}}(t)\right|}^{2}{\kappa}_{p}(0)}.\end{array}$$

The new peak curvature is therefore given by

30 $${\kappa }_{p}(t):= \kappa ({\phi }_{p}(t),t)=\frac{{\left|{{{{{{{\bf{k}}}}}}}}(t)\right|}^{3}}{{\left|{{{{{{{\bf{k}}}}}}}}(t)\right|}^{2}{\left|{{{{{{{\bf{l}}}}}}}}(t)\right|}^{2}-{({{{{{{{\bf{k}}}}}}}}(t)\cdot {{{{{{{\bf{l}}}}}}}}(t))}^{2}}{\kappa }_{p}(0).$$

Since **l** and **k** behave like passive vectors, their dynamics can be described by the deformation tensor

31 $${F}_{ij}(t)=\frac{\partial {X}_{i}({{{{{{{\bf{L}}}}}}}}({\phi }_{0},0),t)}{\partial {x}_{j}},$$

where **X**(**x**, *t*) is the Lagrangian map. The singular value decomposition of *F*,

32 $$F(t)=U(t){{\Lambda }}(t){V}^{T}(t),$$

defines the orthonormal bases ${({{{{{{{{\bf{u}}}}}}}}}_{j}(t))}_{i}={U}_{ij}(t)$ and ${({{{{{{{{\bf{v}}}}}}}}}_{j}(t))}_{i}={V}_{ij}(t)$ and the finite-time Lyapunov exponents *ρ*_*i*_(*t*) by ${{{\Lambda }}}_{ii}={e}^{{\rho }_{i}(t)t}$ where Λ is diagonal. Expanding **l**(0) and **k**(0) in the **v**_*j*_-coordinate system yields

33 $${{{{{{{\bf{l}}}}}}}}(0) 	=\mathop{\sum}\limits_{i}{a}_{i}(t){{{{{{{{\bf{v}}}}}}}}}_{i}(t)\quad \,{{{{{{\rm{and}}}}}}}\,\\ {{{{{{{\bf{k}}}}}}}}(0) 	=\mathop{\sum}\limits_{i}{b}_{i}(t){{{{{{{{\bf{v}}}}}}}}}_{i}(t).$$

Observing that *F*(*t*)**l**(0) = **l**(*t*) and *F*(*t*)**k**(0) = **k**(*t*), and applying the deformation tensor to the previous equations, we get

34 $${{{{{{{\bf{l}}}}}}}}(t) 	=\mathop{\sum}\limits_{i}{a}_{i}(t){e}^{{\rho }_{i}(t)t}{{{{{{{{\bf{u}}}}}}}}}_{i}(t)\quad \,{{{{{{\rm{and}}}}}}}\,\\ {{{{{{{\bf{k}}}}}}}}(t) 	=\mathop{\sum}\limits_{i}{b}_{i}(t){e}^{{\rho }_{i}(t)t}{{{{{{{{\bf{u}}}}}}}}}_{i}(t).$$

Inserting these expansions into (30) yields

35 $${\kappa }_{p}(t)=\frac{{\left({\sum }_{i}{b}_{i}^{2}{e}^{2{\rho }_{i}t}\right)}^{3/2}}{{\sum }_{i\ne j}{a}_{j}{b}_{i}({a}_{j}{b}_{i}-{a}_{i}{b}_{j}){e}^{(2{\rho }_{i}+2{\rho }_{j})t}}{\kappa }_{p}(0).$$

As long as the infinite-time Lyapunov exponents (the *t* → *∞*-limits of the FTLEs) are distinct from each other, we will have ${e}^{{\rho }_{1}(t)t}\gg {e}^{{\rho }_{2}(t)t}\gg {e}^{{\rho }_{3}(t)t}$ for large *t*. Assuming furthermore that the random coefficients in (35) are non-zero, we can drop those terms with slower exponential growth:

36 $${\kappa }_{p}(t)\mathop{\approx }\limits^{t\gg 0}\frac{| {b}_{1}(t){| }^{3}}{{({a}_{2}(t){b}_{1}(t)-{a}_{1}(t){b}_{2}(t))}^{2}}{\kappa }_{p}(0){e}^{[{\rho }_{1}(t)-2{\rho }_{2}(t)]t}.$$

While the FTLEs are known to converge slowly, *V*(*t*) and thus *a*_*i*_(*t*) and *b*_*i*_(*t*) converge exponentially fast^56,57^. We therefore have (cf. ref. ^32^)

37 $${\kappa }_{p}(t)\mathop{\approx }\limits^{t\gg 0}{\widetilde{\kappa }}_{0}{e}^{[{\rho }_{1}(t)-2{\rho }_{2}(t)]t},$$

for some effective initial peak curvature

38 $${\widetilde{\kappa }}_{0}=\mathop{\lim }\limits_{t\to \infty }\frac{| {b}_{1}(t){| }^{3}}{{({a}_{2}(t){b}_{1}(t)-{a}_{1}(t){b}_{2}(t))}^{2}}{\kappa }_{p}(0),$$

which may differ from the actual initial peak curvature *κ*_*p*_(0) depending on the relative orientation of the initial parabola and the converged basis vectors $\mathop{\lim }\nolimits_{t\to \infty }{{{{{{{{\bf{v}}}}}}}}}_{j}(t)$.

### Extracting the power law by the method of steepest descent

In order to extract the asymptotic regime of the integral (14), we substitute the integration variable

39 $${\rho }_{p}=\frac{1}{\tau }\log \left(\frac{{\kappa }_{p}}{{\kappa }_{0}}\right),$$

which yields

40 $$f({\kappa }_{p}) \sim \frac{1}{{\kappa }_{p}}\int\nolimits_{0}^{\infty }{{{{{{{\rm{d}}}}}}}}{\rho }_{p}\frac{N(\log ({\kappa }_{p}/{\kappa }_{0})/{\rho }_{p})}{{\rho }_{p}}\exp\left(-\log \left(\frac{{\kappa }_{p}}{{\kappa }_{0}}\right)\frac{(\beta +S({\rho }_{p}))}{{\rho }_{p}}\right).$$

We now explore the regime where $\log ({\kappa }_{p}/{\kappa }_{0})$ becomes large. Assuming that the normalization function *N*(*τ*) is algebraic, the scaling of the integral with *κ*_*p*_ is dominated by the exponential, and in particular by the part that has the slowest decay. To first order, we therefore have^41, Chapter 9, Theorem 2.1^

41 $$f({\kappa }_{p}) 	\sim \frac{1}{{\kappa }_{p}}\exp \left(-\log \left(\frac{{\kappa }_{p}}{{\kappa }_{0}}\right)\mathop{\min }\limits_{{\rho }_{p}}\left[\frac{1}{{\rho }_{p}}(\beta +S({\rho }_{p}))\right]\right)\\ 	 \propto {\kappa }_{p}^{-1-\alpha },$$

with

42 $$\alpha =\mathop{\min }\limits_{{\rho }_{p}}\left[\frac{1}{{\rho }_{p}}(\beta +S({\rho }_{p}))\right].$$

### Relating our results to generalized Lyapunov exponents

Let us define a generalized Lyapunov exponent of curvature peaks by

43 $${L}_{p}(q)=\mathop{\lim }\limits_{t\to \infty }\frac{1}{t}\log \left\langle \exp (q{\rho }_{p}(t)t)\right\rangle .$$

It differs from the usual definition of generalized Lyapunov exponents only by the fact that we have replaced the standard FTLE by our curvature peak FTLE *ρ*_*p*_(*t*) = *ρ*_1_(*t*) − 2*ρ*_2_(*t*). It is related to the Cramér function by a Legendre transform^39^,

44 $${L}_{p}(q)=\mathop{\sup }\limits_{{\rho }_{p}}\left[q{\rho }_{p}-S({\rho }_{p})\right].$$

This strongly resembles our steepest-descent formula established in the main text (cf. (15)),

45 $$\alpha =\mathop{\min }\limits_{{\rho }_{p}}\left[\frac{1}{{\rho }_{p}}(\beta +S({\rho }_{p}))\right]$$

where, recall, *β* is identified with line growth quantified by the first FTLE (see Fig. 3 and subsequent discussion)

46 $$\beta =\mathop{\lim }\limits_{t\to \infty }\frac{1}{t}\log \left\langle \exp ({\rho }_{1}(t)t)\right\rangle .$$

We claim *L*_*p*_(*α*) = *β*. If so, then equating (43) evaluated at *α* with *β* given by (46), we find

47 $$\mathop{\lim }\limits_{t\to \infty }\frac{1}{t}\log \left\langle \exp (\alpha {\rho }_{p}(t)t)\right\rangle =\mathop{\lim }\limits_{t\to \infty }\frac{1}{t}\log \left\langle \exp ({\rho }_{1}(t)t)\right\rangle ,$$

which we write in short form as (17).

To verify that *L*_*p*_(*α*) = *β*, we insert *α* into (44) to find

48 $${L}_{p}(\alpha )=\mathop{\sup }\limits_{{\rho }_{p}}\left[\alpha {\rho }_{p}-S({\rho }_{p})\right].$$

Assuming that *S*(*ρ*_*p*_) is differentiable and strictly convex, the supremum in (48) occurs at a unique value ${\rho }_{p}^{* }$. Moreover, somewhat remarkably, we will show that this value coincides with that at which the minimum of (45) occurs. Once established, this gives the claimed result upon substitution of $\alpha =\frac{1}{{\rho }_{p}^{* }}(\beta +S({\rho }_{p}^{* }))$ into ${L}_{p}(\alpha )=\alpha {\rho }_{p}^{* }-S({\rho }_{p}^{* })$.

To see that the extrema in (48) and (45) occur at the same point ${\rho }_{p}^{* }$, we note that under our assumptions (48) is minimized at the *ρ* = ρ^*^ for which

49 $$0=\frac{{{{{{{{\rm{d}}}}}}}}}{{{{{{{{\rm{d}}}}}}}}\rho }\left[\alpha \rho -S(\rho )\right]\bigg|_{\rho = {\rho }^{* }}=\alpha -S^{\prime} ({\rho }^{* }).$$

Uniqueness follows from our assumption that $S^{\prime} (\rho )$ is an invertible function of *ρ*. On the other hand, the minimum in (45) occurs for *ρ* = ρ^**^ satisfying

50 $$0 	=\frac{{{{{{{{\rm{d}}}}}}}}}{{{{{{{{\rm{d}}}}}}}}\rho }\left[\frac{1}{\rho }(\beta +S(\rho ))\right]{\bigg| }_{\rho = {\rho }^{* * }}\\ 	=-\frac{1}{{\rho }^{* * }}\left(\frac{1}{{\rho }^{* * }}(\beta +S({\rho }^{* * }))-S^{\prime} ({\rho }^{* * })\right)\\ 	=-\frac{1}{{\rho }^{* * }}\left(\alpha -S^{\prime} ({\rho }^{* * })\right)$$

where we have inserted the expression for *α* in terms of the minimizing argument ρ^**^ given by (45). It is clear from comparing (49) and (50) that the extrema are realized at the same value ${\rho }^{* }={\rho }^{* * }=:{\rho }_{p}^{* }$. This concludes the proof.

### Fokker-Planck equation of curvature in the Kraichnan model

In the Kraichnan model, the velocity field **u**(**x**, *t*) is Gaussian with correlation tensor

51 $$\left\langle {u}_{i}({{{{{{{\bf{x}}}}}}}},t){u}_{j}({{{{{{{\bf{x}}}}}}}}^{\prime} ,t^{\prime} )\right\rangle =\delta (t-t^{\prime} ){R}_{ij}({{{{{{{\bf{x}}}}}}}}-{{{{{{{\bf{x}}}}}}}}^{\prime} ),$$

where *R*_*ij*_(**r**) denotes the spatial part of the correlation tensor.

Equivalent to (3), the curvature PDF weighted by arc length can be defined by

52 $$f(\kappa ;t)=\frac{\left\langle | {\partial }_{\phi }{{{{{{{\bf{L}}}}}}}}| \delta (\kappa -\tilde{\kappa }(\phi ,t))\right\rangle }{\left\langle | {\partial }_{\phi }{{{{{{{\bf{L}}}}}}}}| \right\rangle },$$

where we distinguish between the realization $\tilde{\kappa }$ and the sample-space variable *κ*. Angular brackets 〈·〉 denote an average along *ϕ* and over realizations of the velocity field.

In order to derive the Fokker-Planck equation of curvature, we take the time derivative of (52), which yields

53 $${\partial }_{t}\, f(\kappa ;t)= 	{\,}\frac{\left\langle \delta (\kappa -\tilde{\kappa }){\partial }_{t}| {\partial }_{\phi }{{{{{{{\bf{L}}}}}}}}| \right\rangle }{\left\langle | {\partial }_{\phi }{{{{{{{\bf{L}}}}}}}}| \right\rangle }-f(\kappa ;t)\frac{{\partial }_{t}\left\langle | {\partial }_{\phi }{{{{{{{\bf{L}}}}}}}}| \right\rangle }{\left\langle | {\partial }_{\phi }{{{{{{{\bf{L}}}}}}}}| \right\rangle }\\ 	-\frac{1}{\left\langle | {\partial }_{\phi }{{{{{{{\bf{L}}}}}}}}| \right\rangle }{\partial }_{\kappa }\left\langle \delta (\kappa -\tilde{\kappa })| {\partial }_{\phi }{{{{{{{\bf{L}}}}}}}}| {\partial }_{t}\tilde{\kappa }\right\rangle .$$

The averages can be evaluated using the Gaussian integration by parts formula^58–60^ and the evolution equations^22^

54 $${\partial }_{t}{\partial }_{\phi }{{{{{{{\bf{L}}}}}}}}=(({\partial }_{\phi }{{{{{{{\bf{L}}}}}}}})\cdot \nabla ){{{{{{{\bf{u}}}}}}}},$$

55 $${\partial }_{t}\hat{{{{{{{{\bf{t}}}}}}}}}=(\hat{{{{{{{{\bf{t}}}}}}}}}\cdot \nabla ){{{{{{{\bf{u}}}}}}}}-\hat{{{{{{{{\bf{t}}}}}}}}}(\hat{{{{{{{{\bf{t}}}}}}}}}\cdot (\hat{{{{{{{{\bf{t}}}}}}}}}\cdot \nabla ){{{{{{{\bf{u}}}}}}}}),$$

56 $${\partial }_{t}\hat{{{{{{{{\bf{n}}}}}}}}}=\; \hat{{{{{{{{\bf{b}}}}}}}}}(\hat{{{{{{{{\bf{b}}}}}}}}}\cdot (\hat{{{{{{{{\bf{n}}}}}}}}}\cdot \nabla ){{{{{{{\bf{u}}}}}}}})-\hat{{{{{{{{\bf{t}}}}}}}}}(\hat{{{{{{{{\bf{n}}}}}}}}}\cdot (\hat{{{{{{{{\bf{t}}}}}}}}}\cdot \nabla ){{{{{{{\bf{u}}}}}}}}) +\frac{1}{\tilde{\kappa }}\hat{{{{{{{{\bf{b}}}}}}}}}(\hat{{{{{{{{\bf{b}}}}}}}}}\cdot {(\hat{{{{{{{{\bf{t}}}}}}}}}\cdot \nabla )}^{2}{{{{{{{\bf{u}}}}}}}}),$$

57 $${\partial }_{t}\tilde{\kappa }=\; \tilde{\kappa }\left(\hat{{{{{{{{\bf{n}}}}}}}}}\cdot (\hat{{{{{{{{\bf{n}}}}}}}}}\cdot \nabla ){{{{{{{\bf{u}}}}}}}}-2\hat{{{{{{{{\bf{t}}}}}}}}}\cdot (\hat{{{{{{{{\bf{t}}}}}}}}}\cdot \nabla ){{{{{{{\bf{u}}}}}}}}\right) +\hat{{{{{{{{\bf{n}}}}}}}}}\cdot {(\hat{{{{{{{{\bf{t}}}}}}}}}\cdot \nabla )}^{2}{{{{{{{\bf{u}}}}}}}}.$$

Here, $\hat{{{{{{{{\bf{t}}}}}}}}}$, $\hat{{{{{{{{\bf{n}}}}}}}}}$, and $\hat{{{{{{{{\bf{b}}}}}}}}}$ denote the tangent, normal and binormal vector of the Frenet-Serret frame, respectively. As shown in Supplementary Note 5, the evolution equations derive from the definitions of the various quantities combined with the tracer equation (1). All quantities are evaluated along the same Lagrangian trajectory.

In order to simplify the resulting expressions, we need to further restrict the spatial correlation structure of the model. Isotropy and incompressibility determine the form of the even derivatives of the spatial correlation tensor *R*_*ij*_(**r**) at **0** (odd numbers of derivatives vanish) to be^61^

58 $$-{\partial }_{k}{\partial }_{l}{R}_{ij}({{{{{{{\bf{0}}}}}}}})=Q(4{\delta }_{ij}{\delta }_{kl}-{\delta }_{ik}{\delta }_{jl}-{\delta }_{il}{\delta }_{jk})$$

and^62^

59 $${\partial }_{k}{\partial }_{l}{\partial }_{m}{\partial }_{n}{R}_{ij}({{{{{{{\bf{0}}}}}}}})= P\left(6{\delta }_{ij}{\delta }_{kl}{\delta }_{mn}+6{\delta }_{ij}{\delta }_{km}{\delta }_{ln}+\,6{\delta }_{ij}{\delta }_{kn}{\delta }_{lm}-({{{{\mbox{all others}}}}})\right),$$

with *Q* and *P* scalar constants that depend on the exact form of *R*_*ij*_(**r**). The last pair of brackets contains all 12 other permutations of Kronecker deltas. All terms arising from the Gaussian integration by parts formula can be evaluated using this result and the orthonormality of the Frenet-Serret frame. The resulting Fokker-Planck equation is (20). In Supplementary Note 6, we list results for all terms and exemplify computing one of them.

## Supplementary information

Supplementary Information

Description of additional Supplementary File

Supplementary Movie

## Data Availability

The data that support the findings of this study are available from the corresponding author upon reasonable request.

## Code availability

The simulation results have been generated with our code TurTLE^48^, which is available on https://gitlab.mpcdf.mpg.de/TurTLE/turtle. The loop refinement and post-processing codes are available from the corresponding author upon reasonable request.
