# Supplementary material for: The statistical geometry of material loops in turbulence
Source: Nat Commun. 2022 Apr 19;13:2088. doi: 10.1038/s41467-022-29422-1 (PMC9018957; doi:10.1038/s41467-022-29422-1)
Supplement: Supplementary file 1 — Supplementary Information [file 41467_2022_29422_MOESM1_ESM.pdf]

Supplementary Material for "The statistical geometry of material loops in turbulence"

Lukas Bentkamp, Theodore D. Drivas, Cristian C. Lalescu, and Michael Wilczek

SUPPLEMENTARY NOTE 1. CURVATURE STATISTICS AND DETERMINATION OF POWER-LAW EXPONENT AT VARIOUS REYNOLDS NUMBERS

We carried out our analysis for two additional direct numerical simulations (DNS) of the Navier-Stokes equation, one at Taylor-scale Reynolds number $R_\lambda \approx 147$ and one at $R_\lambda \approx 334$. The simulation details are summarized in Table S1. The curvature statistics of an ensemble of material loops were determined in each simulation as described in Methods. The curvature distributions of the supplementary simulations are shown in Fig. S1. Remarkably, the large-curvature power-law exponents, determined by best fits, are almost the same across the simulations, indicating that there may be no significant Reynolds-number dependence in this range. This becomes even more apparent in Fig. S2a, where the curvature distributions are shown to almost perfectly collapse for the latest point in time in the three simulations when nondimensionalized by η. As a function of the integral length, the distributions shift toward larger κ with increasing Reynolds number (Fig. S2b). Also the curvature peak PDFs in Figure S3 show no measurable Reynolds-number dependence. Consistent with our theory, their high-curvature exponent differs from the curvature PDF exponent by 1, mostly within error bars.

The curvature peak number above different thresholds as a function of time is shown in Figure S4 for the two

	Loops simulations								FTLE simulations						
N	$k_{\max}\eta$	R_λ	$\langle u^2\rangle^{1/2}$	L	L/η	T/τ_η	n_L	$\tau_{\mathrm{ref}}/\tau_\eta$	$k_{\max}\eta$	R_λ	$\langle u^2\rangle^{1/2}$	L	L/η	T/τ_η	n
512	2.0	147	0.96	0.93	94	15.2	250	0.15	2.0	142	0.96	0.91	93	15.4	2.5×10^7
1024	2.9	216	1.09	1.06	148	19.8	1000	0.16	2.9	215	1.09	1.06	148	19.8	1×10^8
2048	2.9	334	1.07	1.04	289	31.2	1000	0.17	3.0	335	1.07	1.04	289	31.1	1×10^9

Supplementary Table S1. **Main DNS Parameters.** Our simulations are run on three-dimensional periodic domains of side length 2π discretized on a real-space grid with N^3 points. The Kolmogorov length and time scales, η and τ_η, respectively, are computed from the mean kinetic energy dissipation ε and the kinematic viscosity ν. Based on the largest wavenumber $k_{\max}$ resolved by our code, we compute the resolution criterion $k_{\max}\eta$. Using the root-mean-squared velocity component $\langle u^2\rangle^{1/2}$ and the energy spectrum $E(k)$, we define the integral length $L = \frac{\pi}{2\langle u^2\rangle}\int \frac{\mathrm{d}k}{k}E(k)$. The integral time scale is computed as $T = L\langle u^2\rangle^{-1/2}$. Although loops and FTLE simulations are initialized with identical fields and parameters, the flows eventually diverge due to numerical rounding errors and chaos. The loops simulations contain n_L material loops, initially sampled by 300 tracer particles per loop whose number increases roughly exponentially due to refinement in intervals of τ_{ref}. For the FTLE simulations, n tracer trajectories are integrated along with the flow field.

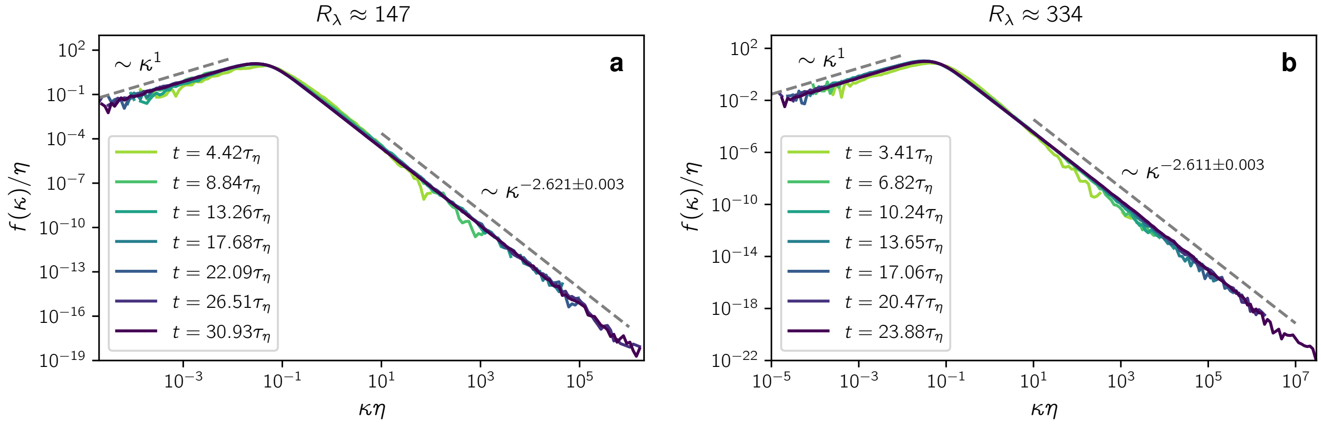

Supplementary Figure S1. **Curvature PDFs in supplementary simulations.** Both in the low-Reynolds-number (**a**) and the high-Reynolds-number (**b**) supplementary simulation, they strongly resemble the distribution of the main simulation. Their power-law exponents, determined by best fits, are almost identical across all simulations.

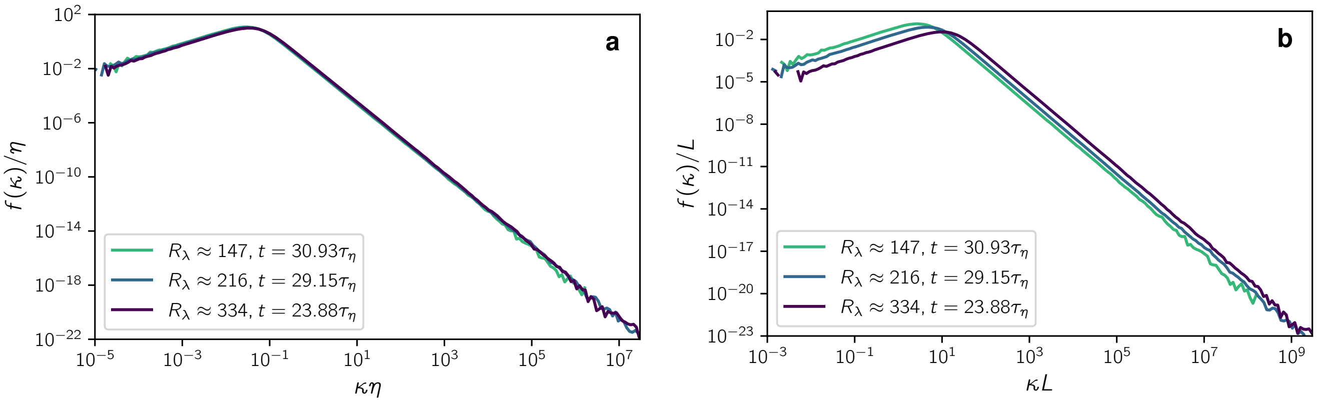

Supplementary Figure S2. **Comparison of curvature PDFs at the latest simulation times**, scaled by the Kolmogorov length (**a**) and the integral length (**b**). In Kolmogorov units, hardly any trend is visible whereas the peak of the PDF shifts toward larger curvature values with increasing Reynolds number when nondimensionalized in integral units.

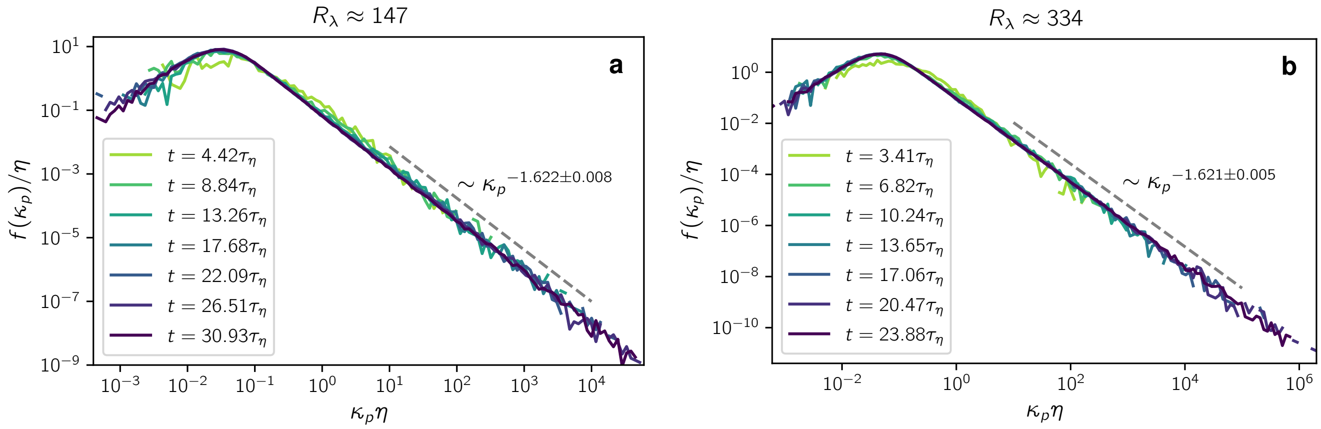

Supplementary Figure S3. **Curvature peak statistics** in the low-Reynolds-number (**a**) and the high-Reynolds-number (**b**) supplementary simulation look the same as in the main simulation. Like for the curvature PDF, the large-curvature power-law exponents, determined by best fits, are almost identical across all simulations.

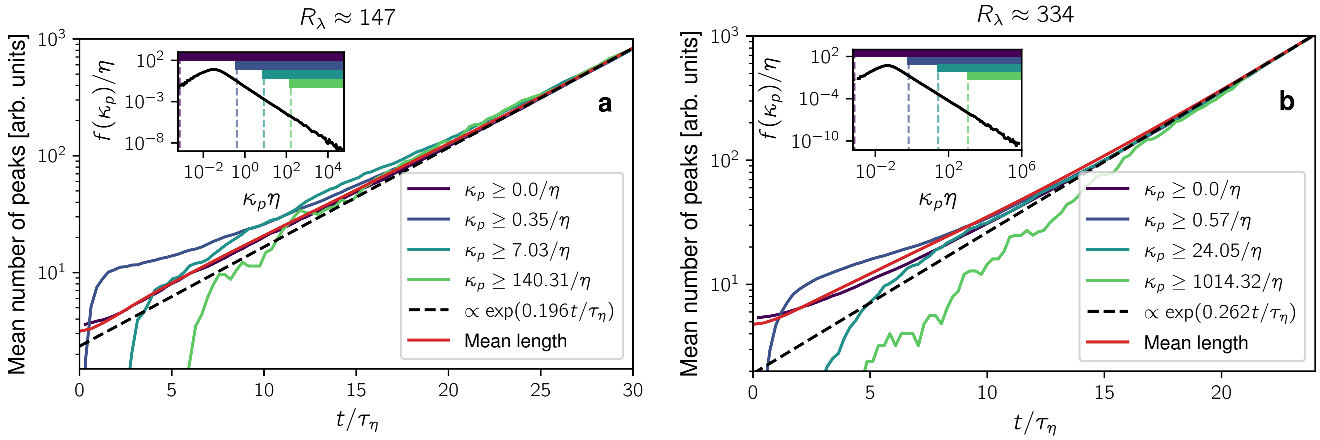

Supplementary Figure S4. **Mean curvature peak number as a function of time** in the low-Reynolds-number (**a**) and the high-Reynolds-number (**b**) supplementary simulation. The mean number of peaks above different thresholds grows exponentially, proportional to the mean length of the loops. Lines are vertically shifted to compare their growth rate, which is why they are not necessarily ordered as a function of the threshold condition. Best fits to the last third of the total peak number curve yield $\beta = (0.19580 \pm 0.00028)/\tau_\eta$ (**a**) and $\beta = (0.26207 \pm 0.00014)/\tau_\eta$ (**b**). As in the main simulation, the standard error from the fit is so small that we neglect it in the following. Insets: Curvature peak distribution at the latest simulation time indicating the different thresholds.

supplementary simulations. Notably, our observation that the curvature peak number grows proportionally to the mean length of the loops carries over to these Reynolds numbers. The corresponding growth rate β in units of the Kolmogorov time appears to increase as a function of Reynolds number.

Finally, we also determine FTLE statistics by integrating the deformation tensor along trajectories of randomly distributed particles. This is done for 25 million tracer particles in an additional simulation at the smaller Reynolds number and for one billion tracer particles in an additional simulation at the larger Reynolds number that use the same initial condition as the corresponding loops simulations. In Figure S5, we determine the steepest-descent minima needed for our theoretical prediction. Notice that the minimum is taken at values of ρ_p that increase with Reynolds number and therefore reach further into the tail of the FTLE distribution. Hence in the large-Reynolds-number simulation, despite the enormous number of tracer particles tracked, the minimum can only be resolved up to $t \approx 25\tau_\eta$. As in the main simulation, we determine the asymptotic value of the minimum by fitting the algebraic decay function (24) to those data points with $t \geq t_{\min}$ (Figure S5, insets). The time $t_{\min}$ is chosen based on the weighted mean squared error, as explained in Figure S6, in order to filter out a transient regime of the decay. The resulting exponents are $\alpha = A = 0.61 \pm 0.08$ for the low-Reynolds-number simulation, $\alpha = A = 0.54 \pm 0.11$ for the main simulation and $\alpha = A = 0.55 \pm 0.07$ for the high-Reynolds-number simulation, all of them consistent with the measured curvature PDF power-law exponents.

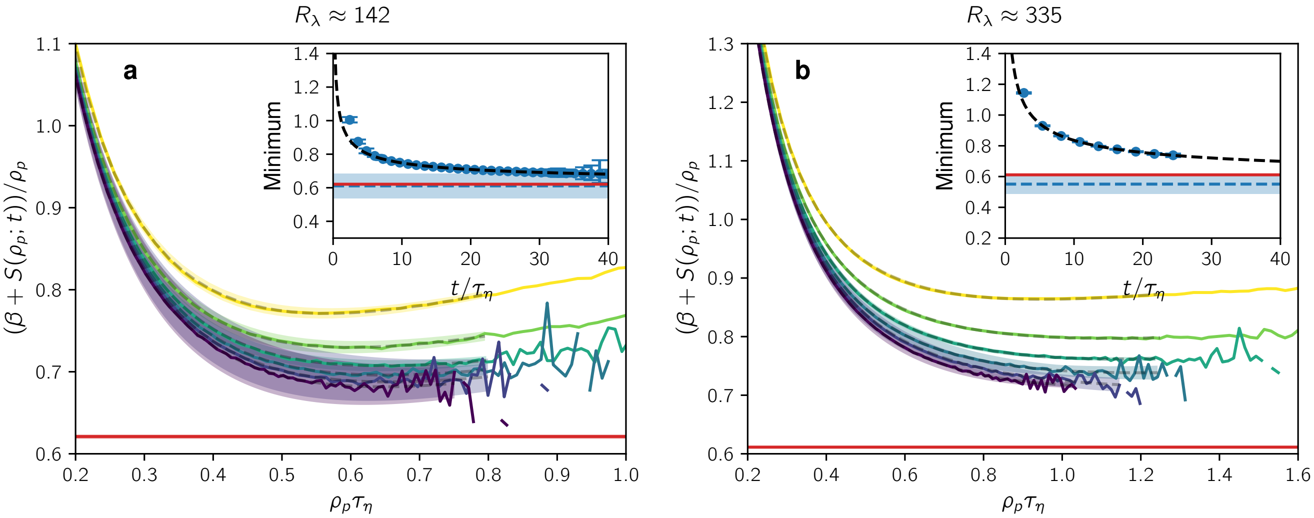

Supplementary Figure S5. **Determination of the steepest-descent minimum for the supplementary simulations. a** Low-Reynolds-number simulation with Cramér functions ranging from $7.26\tau_\eta$ (yellow) up to $39.94\tau_\eta$ (violet). Their fits are restricted to the range $[\lambda_p(t), 0.8/\tau_\eta]$, where $\lambda_p(t)$ is the position of the minimum of $S(\rho_p;t)$. Inset: Extrapolation of the minimum yields $\alpha = 0.61 \pm 0.08$ (dashed blue line). **b** High-Reynolds-number simulation with Cramér functions at times ranging from $8.14\tau_\eta$ (yellow) up to $35.26\tau_\eta$ (violet). Their fits are restricted to the range $[\lambda_p(t), 1.25/\tau_\eta]$. Inset: Extrapolation of the minimum yields $\alpha = 0.55 \pm 0.07$ (dashed blue line). For comparison, in each plot the red line indicates the value of α estimated from the curvature PDF. For details on the procedure, see Methods.

SUPPLEMENTARY NOTE 2. NUMERICAL ANALYSIS OF GENERALIZED LYAPUNOV EXPONENTS

As a complementary approach to computing Cramér functions, we may also use generalized Lyapunov exponents (GLE) in order to determine the exponent α based on the implicit equation (47), in short: $L_p(\alpha) = \beta = L_1(1)$, where

$$L_1(q) = \lim_{t\to\infty} \frac{1}{t} \log \left\langle \exp(q\rho_1(t)t) \right\rangle \tag{S1}$$

is the first standard GLE as opposed to

$$L_p(q) = \lim_{t\to\infty} \frac{1}{t} \log \left\langle \exp(q\rho_p(t)t) \right\rangle , \tag{S2}$$

the curvature-peak GLE. For their numerical computation, we adopt the method from ref. [1]. We first compute the cumulant-generating function of $\rho_1(t)t$, given by $\log\langle \exp(q\rho_1(t)t)\rangle$, as a function of q and t. In order to estimate

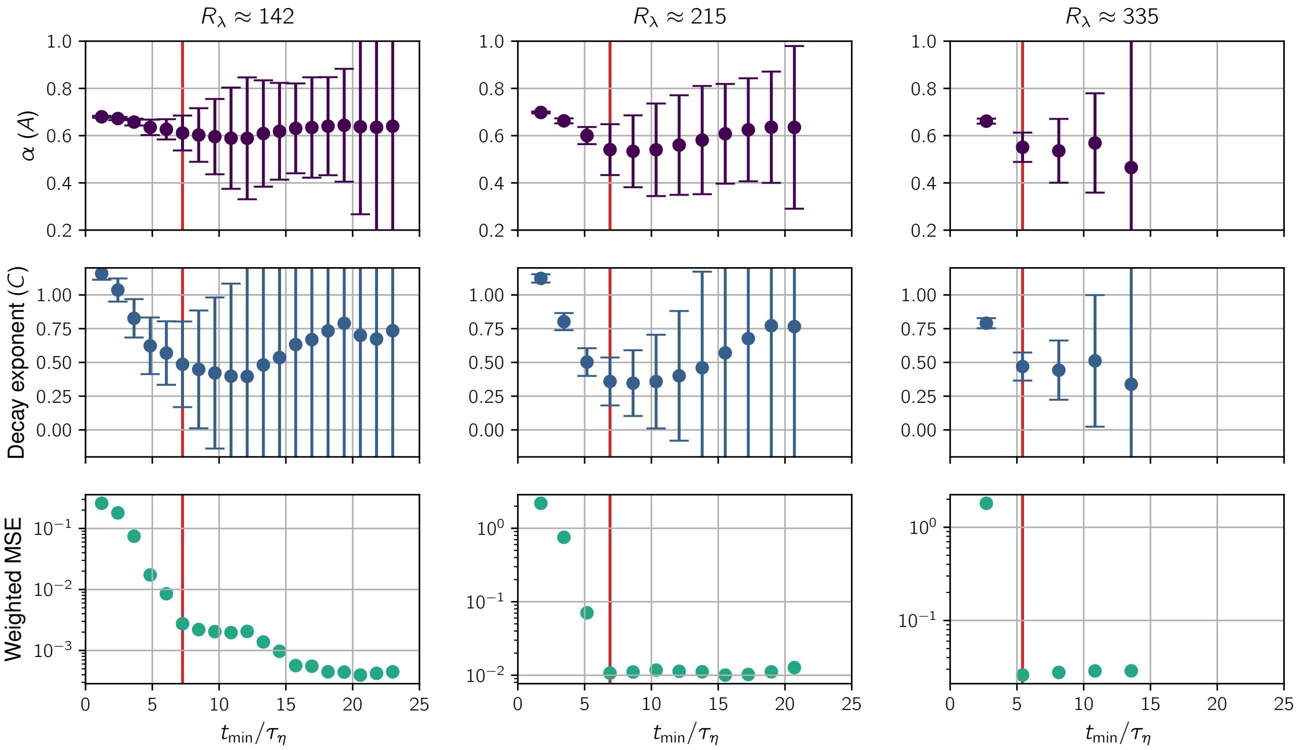

Supplementary Figure S6. **Different fit choices for extrapolating the steepest-descent minimum.** In order to compute the steepest-descent minimum (15), in principle we need a fully resolved Cramér function. In practice, however, we only have finite-time estimates of the Cramér function, which yield finite-time estimates of the minimum (our "data points" in the insets of Figs. 5 and S5). We use the simple decay function (24) to capture and extrapolate the evolution of these data points as a function of the time at which the finite-time Cramér function is computed. In order to obtain a satisfactory fit, it is helpful to leave out a transient regime of data points $t < t_{\min}$ such that only the asymptotic behavior is captured by the fit. Here we show the resulting values of $\alpha = A$ (top) and the decay exponent C (middle) for different choices of $t_{\min}$ in all of the simulations (left to right). The time scale B is not shown. We justify the choice of $t_{\min}$ by computing the weighted mean squared error (MSE, bottom) given by $\sum_{i=1}^{N}(\delta_i/\sigma_i)^2/(N-3)$. Here, N is the number of data points included, δ_i is the deviation of the fit from the i-th data point, and σ_i is the error of the i-th data point, given by the maximum of the two-sided error computed from the error envelopes in Figs. 5 and S5. The quantity $\sum_{i=1}^{N}(\delta_i/\sigma_i)^2$ is minimized by the fit, which we divide by the number of degrees of freedom $N-3$ (number of data points minus number of fit parameters) for comparability. We choose $t_{\min}$ to be the start of the first plateau of the weighted MSE (red lines).

$L_1(q)$, we perform an affine fit of the cumulant-generating function in the range $t \in [t_{\max}/2, t_{\max}]$, as exemplified in Fig. S7a for $t_{\max} = 40\tau_\eta$. The slope of each fit including its standard error becomes our estimate of $L_1(q)$. The same procedure is applied to $L_p(q)$.

For the main simulation, the results are shown in Fig. S7b. We can first read off the value of β by evaluating $L_1(q)$ at $q = 1$. Indeed, the different estimates for $L_1(1)$ appear to converge toward the value of β previously estimated by other means. In order to estimate α, we need to read off the intersection of the β-line with $L_p(q)$. For this curvature-peak GLE, we observe stronger fluctuations as a function of $t_{\max}$. For small $t_{\max}$, we expect the estimates of the cumulant-generating function to be accurate. However, if $t_{\max}$ is too small, we have not yet reached convergence of the $t \to \infty$ limit in the GLE. For larger $t_{\max}$, we improve on the convergence of the GLE, but we also rely more heavily on extreme values of $\rho_p(t)$ (especially for large q), which are limited by our sample size. Hence we expect the best estimate to be found at intermediate $t_{\max}$. For the main simulation, we indeed find those intermediate curves to come closest to the value of α estimated from the curvature PDF. For the supplementary simulations (Fig. S8), the same argumentation holds and the estimates fluctuate as a function of $t_{\max}$ to a certain extent. If we simply read off α from the intersection of lines, the GLE method slightly overestimates α for all simulations, possibly due to both sampling and time-convergence limitations.

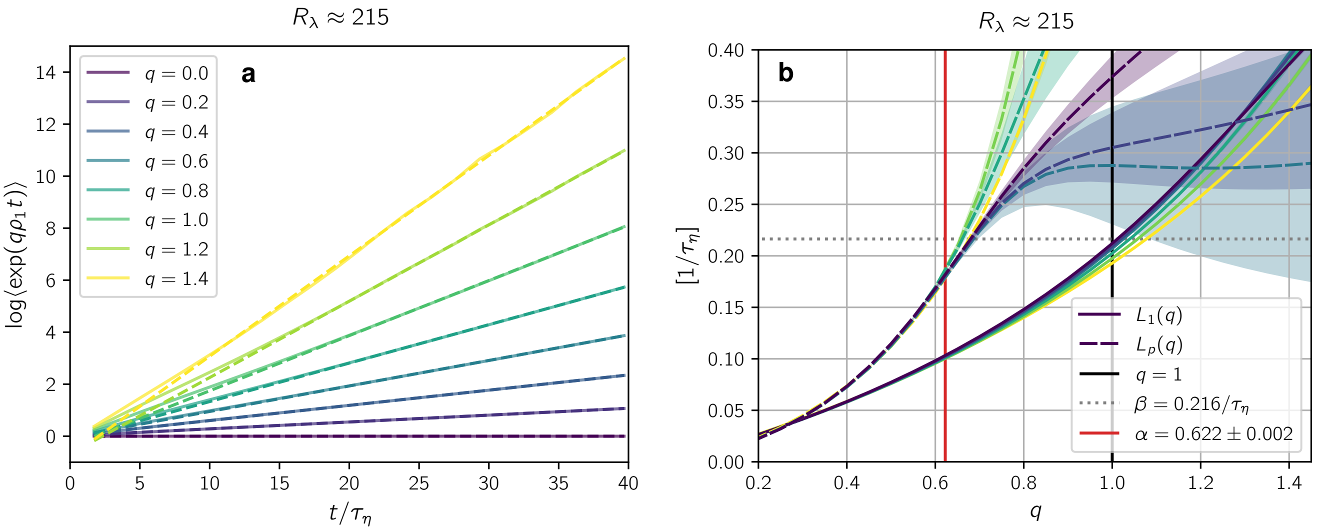

Supplementary Figure S7. **Generalized Lyapunov exponents in the main simulation.** **a** Plotting the cumulant-generating function of $\rho_1(t)t$ for fixed argument q as a function of time (solid lines), the GLE $L_1(q)$ can be estimated as the asymptotic slope of the curve by an affine fit (dashed lines) on the interval $t \in [t_{\max}/2, t_{\max}]$ where in this case $t_{\max} = 40\tau_\eta$. **b** Generalized Lyapunov exponents can be used to estimate β and α. The first standard GLE $L_1(q)$ (solid lines) is shown for $t_{\max}$ ranging from $12.08\tau_\eta$ (yellow) to $39.68\tau_\eta$ (violet). The curvature-peak GLE $L_p(q)$ is shown for the same times (dashed lines). For comparison, we also show the line $q = 1$ (solid, black), the value of β estimated from curvature peak number (dotted, grey) and the value of α estimated from the curvature PDF (solid, red).

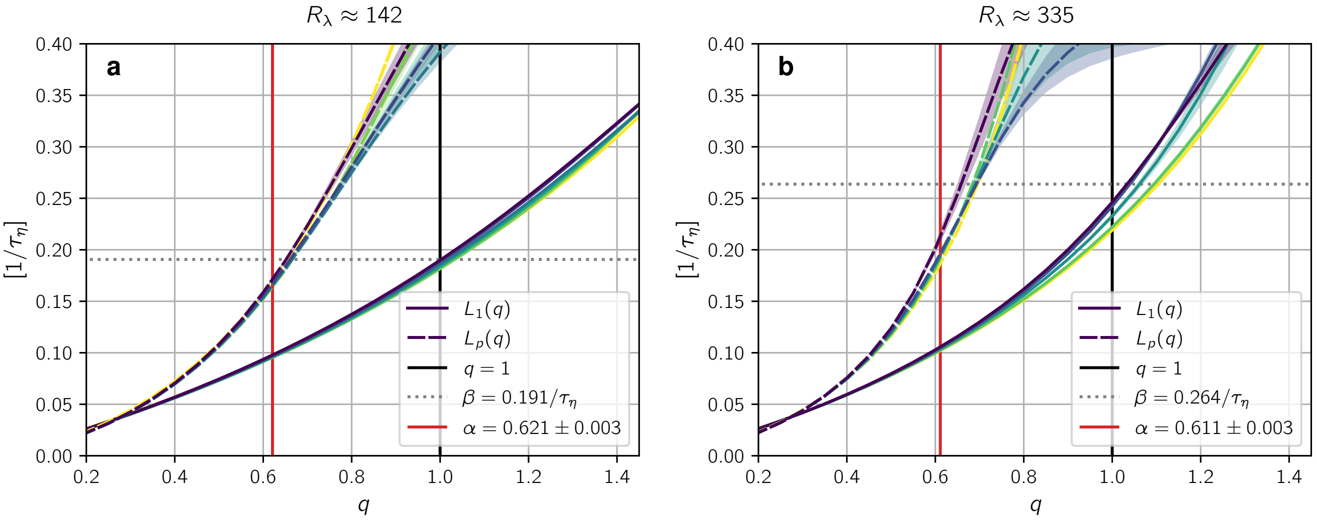

Supplementary Figure S8. **Generalized Lyapunov exponents in the supplementary simulations**, at low Reynolds number (**a**) and at high Reynolds number (**b**). As in the main simulation, the first standard GLE $L_1(1)$ converges toward the previously estimated value of β, but the convergence is slower in the high-Reynolds-number simulation. Similarly, the value of α is slightly overestimated in both simulations. In **a**, $t_{\max}$ ranges from $12.10\tau_\eta$ (yellow) to $39.94\tau_\eta$ (violet). In **b**, $t_{\max}$ ranges from $16.27\tau_\eta$ (yellow) to $35.26\tau_\eta$ (violet). Note that the value of β as a function of the Kolmogorov time scale τ_η differs from the one in Fig. S4 because τ_η is slightly different in the FTLE simulation.

SUPPLEMENTARY NOTE 3. MATERIAL LINE BUNDLES AND FLUX CANCELLATIONS

In the discussion, we explain how our results may help to shed light on the problem of flux cancellations in magnetohydrodynamics (MHD). Flux cancellations occur when magnetic field lines with opposite orientation are brought closely together by the flow and thus cancel each other in the integration of magnetic flux. In our simulations, we observe that the material lines are brought into such a configuration quite frequently (see Fig. S9). They even tend to form bundles of lines where about half of the lines has opposite orientation. Remarkably, these bundles appear to be strongly related to the sharp folds that lead to curvature peaks since the folds are typically observed in the middle or at the end of such bundles. Therefore, it may be worthwhile studying folds of the magnetic field in MHD (possibly detected by curvature peaks) and their relation to flux cancellations.

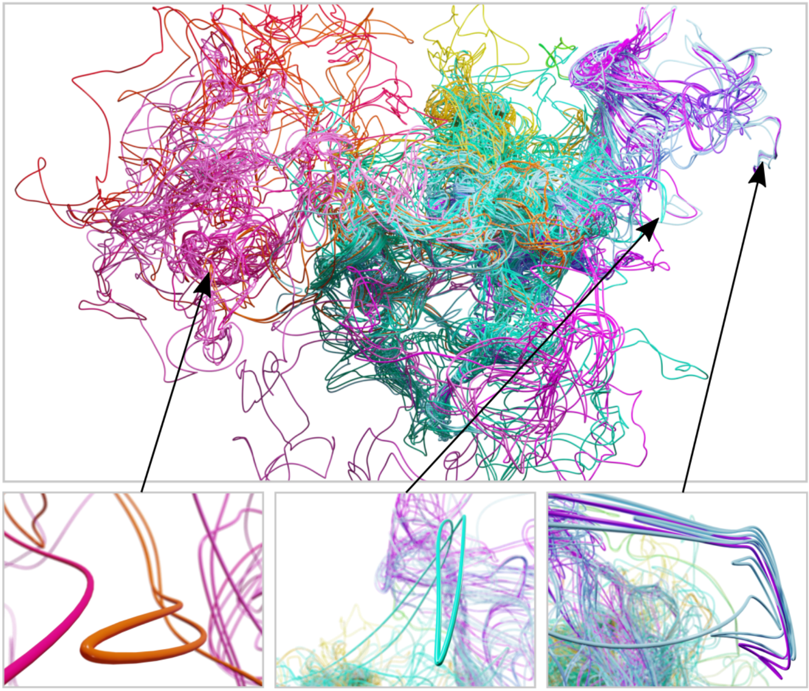

Supplementary Figure S9. **Loops form pairs and bundles of almost parallel material lines**, which are closely associated with sharp folds. The loop snapshot is identical to Fig. 1 of the manuscript, taken at $t = 27\tau_\eta$ in the main simulation at $R_\lambda \approx 216$.

SUPPLEMENTARY NOTE 4. SPATIAL AND TEMPORAL RESOLUTION OF LOOP TRACKING

For tracking material loops in our simulations, we treated the sample points of the loops as Lagrangian tracers. Over time, new particles are inserted to sustain the necessary loop resolution. Since using higher-order time-stepping methods for the particles would require histories that are not available for newly inserted particles, we resorted to first-order Euler time stepping. In order to ensure the quality of our results, we tested the code with different temporal resolutions of flow and particle time stepping and different spatial resolution conditions of the loops. The results for simulations at $R_\lambda \approx 146$ are shown in Fig. S10 where we compare curvature statistics for the different resolutions. We observe that neither an improved temporal resolution nor an improved spatial resolution of the loops leads to noticeable changes in the curvature distribution.

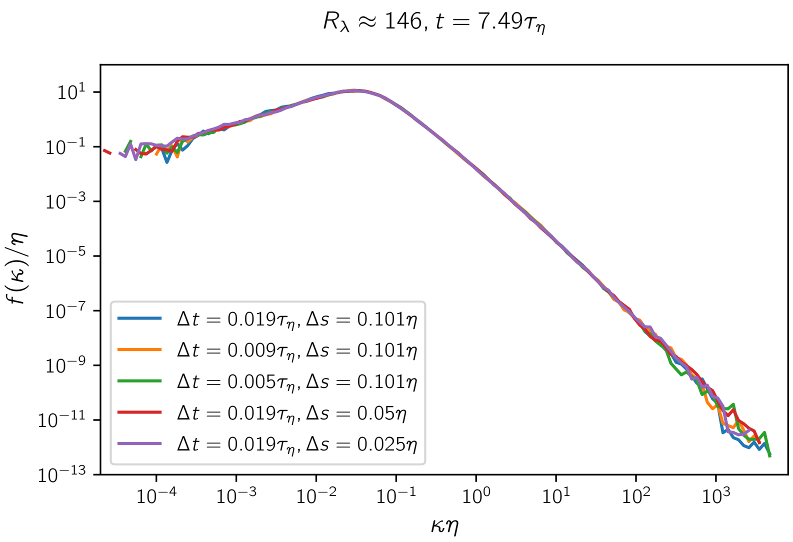

Supplementary Figure S10. **Curvature distributions for different temporal and spatial resolutions of the loops**, taken from 1000 loops after $7.49\tau_\eta$ in simulations at $R_\lambda \approx 146$. Δt is the time-step size of the simulation, including field and particle time stepping. Δs denotes the maximum distance of sampling points of the loops enforced by the refinement. We adjusted the additional refinement condition based on curvature accordingly, effectively doubling the density of sample points uniformly along the loops when moving from $\Delta s = 0.1\eta$ to $\Delta s = 0.05\eta$ and further to $\Delta s = 0.025\eta$. Other parameters are identical with the low-Reynolds-number simulation shown in Supplementary Note 1, which uses $\Delta t = 0.018\tau_\eta$ and $\Delta s = 0.1\eta$. Note that the curved shape of the distribution can be attributed to the early time ($t = 7.49\tau_\eta$) in the loops' evolution.

SUPPLEMENTARY NOTE 5. GEOMETRIC EVOLUTION EQUATIONS

Here, we derive the evolution equations (54)–(57). Implicitly, all quantities considered in these equations are evaluated along a material line element $\mathbf{L} = \mathbf{L}(\phi, t)$, whose evolution is given by the tracer equation (1). Therefore

$$\partial_t \partial_\phi \mathbf{L}(\phi, t) = ((\partial_\phi \mathbf{L}) \cdot \nabla)\mathbf{u}(\mathbf{L}(\phi, t), t) \tag{S3}$$

which is (54). The evolution equation (55) for the tangent vector of the Frenet-Serret frame [2],

$$\hat{\mathbf{t}}(\phi, t) = \frac{\partial_\phi \mathbf{L}}{|\partial_\phi \mathbf{L}|}, \tag{S4}$$

can then be directly computed

$$\partial_t \hat{\mathbf{t}}(\phi, t) = \partial_t \frac{\partial_\phi \mathbf{L}}{|\partial_\phi \mathbf{L}|} = \frac{1}{|\partial_\phi \mathbf{L}|}(\partial_\phi \mathbf{L} \cdot \nabla)\mathbf{u} - \frac{\partial_\phi \mathbf{L}}{|\partial_\phi \mathbf{L}|^3}\partial_\phi \mathbf{L} \cdot (\partial_\phi \mathbf{L} \cdot \nabla)\mathbf{u} = (\hat{\mathbf{t}} \cdot \nabla)\mathbf{u} - (\hat{\mathbf{t}} \cdot (\hat{\mathbf{t}} \cdot \nabla)\mathbf{u})\hat{\mathbf{t}}. \tag{S5}$$

The normal vector of the Frenet-Serret frame is defined as

$$\hat{\mathbf{n}}(\phi, t) = \frac{\partial_s \hat{\mathbf{t}}}{|\partial_s \hat{\mathbf{t}}|}, \tag{S6}$$

where s denotes an arc-length parameterization of the line, i.e. $|\partial_s \mathbf{L}| = 1$. Since the transform from s to ϕ is time-dependent, evaluating ∂_t at constant ϕ and at constant s are different operations. Here, we always want to take ∂_t at constant ϕ (i.e. at the same tracer particle). Then, ∂_s and ∂_t do not commute. Having this in mind, we compute (summation over repeated indices implied)

$$\begin{aligned}\partial_t \hat{n}_i &= \partial_t \frac{\partial_s \hat{t}_i}{|\partial_s \hat{\mathbf{t}}|} = \frac{(\partial_t \partial_s \hat{t}_i)|\partial_s \hat{\mathbf{t}}| - (\partial_s \hat{t}_i)\partial_t |\partial_s \hat{\mathbf{t}}|}{|\partial_s \hat{\mathbf{t}}|^2} = \frac{\partial_t \partial_s \hat{t}_i}{|\partial_s \hat{\mathbf{t}}|} - \hat{n}_i \frac{\partial_t |\partial_s \hat{\mathbf{t}}|}{|\partial_s \hat{\mathbf{t}}|} = \frac{\partial_t \partial_s \hat{t}_i}{|\partial_s \hat{\mathbf{t}}|} - \hat{n}_i \hat{n}_j \frac{1}{|\partial_s \hat{\mathbf{t}}|}\partial_t \partial_s \hat{t}_j \\ &= \frac{1}{|\partial_s \hat{\mathbf{t}}|}\left(\delta_{ij} - \hat{n}_i \hat{n}_j\right) \partial_t \partial_s \hat{t}_j. \end{aligned} \tag{S7}$$

In order to swap the t- and s-derivatives, we notice that $\partial_s = \frac{\mathrm{d}\phi}{\mathrm{d}s}\partial_\phi = \frac{1}{|\partial_\phi \mathbf{L}|}\partial_\phi$. Therefore

$$\partial_t \partial_s \hat{t}_j = \left(\partial_t \frac{1}{|\partial_\phi \mathbf{L}|}\right) \partial_\phi \hat{t}_j + \partial_s \partial_t \hat{t}_j = -\hat{t}_k \hat{t}_l (\partial_k u_l)(\partial_s \hat{t}_j) + \partial_s \partial_t \hat{t}_j. \tag{S8}$$

We then insert the evolution equation (S5) for the tangent vector $\hat{\mathbf{t}}$,

$$\begin{aligned}\partial_t \partial_s \hat{t}_j &= -\hat{t}_k \hat{t}_l (\partial_k u_l)(\partial_s \hat{t}_j) + \partial_s \Big((\delta_{jk} - \hat{t}_j \hat{t}_k)\hat{t}_l \partial_l u_k\Big) \\ &= -\hat{t}_k \hat{t}_l (\partial_k u_l)(\partial_s \hat{t}_j) - \Big((\partial_s \hat{t}_j)\hat{t}_k + \hat{t}_j(\partial_s \hat{t}_k)\Big)\hat{t}_l \partial_l u_k + (\delta_{jk} - \hat{t}_j \hat{t}_k)\Big((\partial_s \hat{t}_l)\partial_l u_k + \hat{t}_l \partial_s \partial_l u_k\Big) \\ &= |\partial_s \hat{\mathbf{t}}|\Big[-\hat{t}_k \hat{t}_l \hat{n}_j (\partial_k u_l) - (\hat{n}_j \hat{t}_k + \hat{t}_j \hat{n}_k)\hat{t}_l \partial_l u_k + (\delta_{jk} - \hat{t}_j \hat{t}_k)\Big(\hat{n}_l \partial_l u_k + \frac{1}{|\partial_s \hat{\mathbf{t}}|}\hat{t}_l \hat{t}_m \partial_l \partial_m u_k\Big)\Big], \end{aligned} \tag{S9}$$

where in the last step we used (S6) and $\partial_s u_k = \hat{t}_m \partial_m u_k$. Since $|\partial_s \hat{\mathbf{t}}| = \tilde{\kappa}$ and $\delta_{ij} = \hat{t}_i \hat{t}_j + \hat{n}_i \hat{n}_j + \hat{b}_i \hat{b}_j$, we have

$$\begin{aligned}\partial_t \hat{n}_i &= (\hat{t}_i \hat{t}_j + \hat{b}_i \hat{b}_j)\Big[-\hat{t}_k \hat{t}_l \hat{n}_j (\partial_k u_l) - (\hat{n}_j \hat{t}_k + \hat{t}_j \hat{n}_k)\hat{t}_l \partial_l u_k + (\hat{n}_j \hat{n}_k + \hat{b}_j \hat{b}_k)\Big(\hat{n}_l \partial_l u_k + \frac{1}{\tilde{\kappa}}\hat{t}_l \hat{t}_m \partial_l \partial_m u_k\Big)\Big] \\ &= -\hat{t}_i \hat{n}_k \hat{t}_l \partial_l u_k + \hat{b}_i \hat{b}_k \hat{n}_l \partial_l u_k + \frac{1}{\tilde{\kappa}}\hat{b}_i \hat{b}_k \hat{t}_l \hat{t}_m \partial_l \partial_m u_k, \end{aligned} \tag{S10}$$

where we also used orthonormality of the Frenet-Serret frame. This is (56).

Finally, in order to derive the curvature evolution equation, we use a simple definition as a function of the tangent vector with arc-length parameterization,

$$\tilde{\kappa}(\phi, t) = |\partial_s \hat{\mathbf{t}}|, \tag{S11}$$

which is equivalent to definition (2). Using the previous results, we obtain

$$\begin{aligned}\partial_t \tilde{\kappa} &= \partial_t |\partial_s \hat{\mathbf{t}}| = \hat{n}_j \partial_t \partial_s \hat{t}_j = \hat{n}_j \tilde{\kappa}\Big[-\hat{t}_k \hat{t}_l \hat{n}_j (\partial_k u_l) - (\hat{n}_j \hat{t}_k + \hat{t}_j \hat{n}_k)\hat{t}_l \partial_l u_k + (\hat{n}_j \hat{n}_k + \hat{b}_j \hat{b}_k)\Big(\hat{n}_l \partial_l u_k + \frac{1}{|\partial_s \hat{\mathbf{t}}|}\hat{t}_l \hat{t}_m \partial_l \partial_m u_k\Big)\Big] \\ &= \tilde{\kappa}\Big(-2\hat{t}_k \hat{t}_l + \hat{n}_k \hat{n}_l\Big)\partial_k u_l + \hat{n}_k \hat{t}_l \hat{t}_m \partial_l \partial_m u_k. \end{aligned} \tag{S12}$$

This is (57), which can also be found in ref. [3].

SUPPLEMENTARY NOTE 6. FOKKER-PLANCK EQUATION IN THE KRAICHNAN MODEL

In Methods, we laid out the terms that need to be calculated in order to arrive at the Fokker-Planck equation in the Kraichnan model. In order to proceed, we combine (53) with the evolution equations derived in the previous section and get

$$\partial_t f(\kappa;t) = \frac{\langle \delta(\kappa-\tilde\kappa)\partial_t|\partial_\phi \mathbf{L}|\rangle}{\langle|\partial_\phi \mathbf{L}|\rangle} - f(\kappa;t)\frac{\partial_t\langle|\partial_\phi \mathbf{L}|\rangle}{\langle|\partial_\phi \mathbf{L}|\rangle} - \frac{1}{\langle|\partial_\phi \mathbf{L}|\rangle}\partial_\kappa\langle\delta(\kappa-\tilde\kappa)|\partial_\phi \mathbf{L}|\partial_t\tilde\kappa\rangle \tag{S13}$$

$$= \frac{1}{\langle|\partial_\phi \mathbf{L}|\rangle}\Bigg(\Big\langle|\partial_\phi \mathbf{L}|\delta(\kappa-\tilde\kappa)\hat t_i\hat t_j\partial_j u_i\Big\rangle - f(\kappa;t)\Big\langle|\partial_\phi \mathbf{L}|\hat t_i\hat t_j\partial_j u_i\Big\rangle \tag{S14}$$

$$- \partial_\kappa\Big(-2\kappa\Big\langle|\partial_\phi \mathbf{L}|\delta(\kappa-\tilde\kappa)\hat t_i\hat t_j\partial_j u_i\Big\rangle + \kappa\Big\langle|\partial_\phi \mathbf{L}|\delta(\kappa-\tilde\kappa)\hat n_i\hat n_j\partial_j u_i\Big\rangle + \Big\langle|\partial_\phi \mathbf{L}|\delta(\kappa-\tilde\kappa)\hat n_i\hat t_j\hat t_k\partial_j\partial_k u_i\Big\rangle\Big)\Bigg).$$

We want to evaluate these averages using the Gaussian integration by parts formula [4–6] combined with the correlation tensor (51). This works analogously for all of them. So let us focus on one of the averages. By introducing delta functions, we can consider the velocity field at the Eulerian coordinate $\mathbf{x}$ and take the derivative out of the average (the other quantities are still evaluated at $\mathbf{L}(\phi,t)$),

$$\langle|\partial_\phi \mathbf{L}|\delta(\kappa-\tilde\kappa)\hat n_i\hat n_j\partial_j u_i\rangle = \int \mathrm{d}^3\mathbf{x}\int \mathrm{d}^3\mathbf{y}\;\delta(\mathbf{x}-\mathbf{y})\,\frac{\partial}{\partial x_j}\langle|\partial_\phi \mathbf{L}|\delta(\mathbf{y}-\mathbf{L}(\phi,t))\delta(\kappa-\tilde\kappa)\hat n_i\hat n_j u_i(\mathbf{x},t)\rangle\,. \tag{S15}$$

Then Gaussian integration by parts yields

$$\langle|\partial_\phi \mathbf{L}|\delta(\kappa-\tilde\kappa)\hat n_i\hat n_j\partial_j u_i\rangle = \int \mathrm{d}^3\mathbf{x}\int \mathrm{d}^3\mathbf{y}\;\delta(\mathbf{x}-\mathbf{y})\,\frac{\partial}{\partial x_j}\int \mathrm{d}^3\mathbf{z}\;R_{ik}(\mathbf{x}-\mathbf{z})\left\langle\frac{\delta\left[|\partial_\phi \mathbf{L}|\delta(\mathbf{y}-\mathbf{L}(\phi,t))\delta(\kappa-\tilde\kappa)\hat n_i\hat n_j\right]}{\delta u_k(\mathbf{z},t)}\right\rangle$$

$$= \int \mathrm{d}^3\mathbf{x}\int \mathrm{d}^3\mathbf{z}\;\big(\partial_j R_{ik}(\mathbf{x}-\mathbf{z})\big)\left\langle\frac{\delta\left[|\partial_\phi \mathbf{L}|\delta(\mathbf{x}-\mathbf{L}(\phi,t))\delta(\kappa-\tilde\kappa)\hat n_i\hat n_j\right]}{\delta u_k(\mathbf{z},t)}\right\rangle. \tag{S16}$$

The product rule for the functional derivative yields five different terms, which can all be treated in the same way. Let us again focus on a single one of them, namely

$$M = \int \mathrm{d}^3\mathbf{x}\int \mathrm{d}^3\mathbf{z}\;\big(\partial_j R_{ik}(\mathbf{x}-\mathbf{z})\big)\left\langle|\partial_\phi \mathbf{L}|\delta(\mathbf{x}-\mathbf{L}(\phi,t))\hat n_i\hat n_j\frac{\delta\left[\delta(\kappa-\tilde\kappa)\right]}{\delta u_k(\mathbf{z},t)}\right\rangle$$

$$= \int \mathrm{d}^3\mathbf{z}\;\left\langle\big(\partial_j R_{ik}(\mathbf{L}(\phi,t)-\mathbf{z})\big)\,|\partial_\phi \mathbf{L}|\hat n_i\hat n_j\delta'(\tilde\kappa-\kappa)\frac{\delta\tilde\kappa}{\delta u_k(\mathbf{z},t)}\right\rangle. \tag{S17}$$

In order to determine the response function $\frac{\delta\tilde\kappa}{\delta u_k(\mathbf{z},t)}$, we formally integrate the curvature evolution equation (S12),

$$\tilde\kappa(\phi,t) = \tilde\kappa(\phi,0) + \int_0^t \mathrm{d}t'\;\Big(\tilde\kappa\hat n_m\hat n_n\partial_n u_m - 2\tilde\kappa\hat t_m\hat t_n\partial_n u_m + \hat n_m\hat t_n\hat t_o\partial_n\partial_o u_m\Big)\Big|_{(\mathbf{L}(\phi,t'),t')}. \tag{S18}$$

By causality, the initial condition will not depend on $u_k(\mathbf{z},t)$ for $t>0$. The integrand will not depend on $u_k(\mathbf{z},t)$ for all $t'<t$ either, and the only contribution to the functional derivative can come from the time $t'=t$. Since $\tilde\kappa$, $\hat{\mathbf{n}}$, and $\hat{\mathbf{t}}$ are integrated quantities of the delta-correlated field $\mathbf{u}$, we expect them to be continuous in time, just like a Wiener process is continuous while its differential is not. Hence their response functions will only be finite, thus not contribute to the integral. Using that

$$\frac{\delta u_m(\mathbf{L}(\phi,t'),t')}{\delta u_k(\mathbf{z},t)} = \delta(\mathbf{L}(\phi,t')-\mathbf{z})\delta(t-t')\delta_{mk}, \tag{S19}$$

the response function becomes

$$\frac{\delta\tilde\kappa(\phi,t)}{\delta u_k(\mathbf{z},t)} = \frac{1}{2}\Big(\tilde\kappa\hat n_k\hat n_n\partial_n\delta(\mathbf{L}(\phi,t)-\mathbf{z}) - 2\tilde\kappa\hat t_k\hat t_n\partial_n\delta(\mathbf{L}(\phi,t)-\mathbf{z}) + \hat n_k\hat t_n\hat t_o\partial_n\partial_o\delta(\mathbf{L}(\phi,t)-\mathbf{z})\Big),$$

where the factor $\frac{1}{2}$ comes from the fact that only half of the delta function $\delta(t-t')$ is contained in the integration range $[0,t]$. Using integration by parts and the sifting property of the delta function, our term M can thus be simplified to

$$M = \frac{1}{2}\partial_\kappa\Bigg(\left(\partial_j\partial_n R_{ik}(\mathbf{0})\right)\kappa\left\langle|\partial_\phi\mathbf{L}|\hat{n}_i\hat{n}_j\delta(\kappa-\tilde{\kappa})(\hat{n}_k\hat{n}_n-2\hat{t}_k\hat{t}_n)\right\rangle - \left(\partial_j\partial_n\partial_o R_{ik}(\mathbf{0})\right)\left\langle|\partial_\phi\mathbf{L}|\hat{n}_i\hat{n}_j\delta(\kappa-\tilde{\kappa})\hat{n}_k\hat{t}_n\hat{t}_o\right\rangle\Bigg).$$

Although the spatial correlation tensor $R_{ik}(\mathbf{r})$ can be freely chosen, we can restrict its functional form by assuming isotropy and incompressibility of the Kraichnan field. By isotropy the correlation tensor must be even, hence odd derivatives vanish at zero, e.g. $\partial_j\partial_n\partial_o R_{ik}(\mathbf{0}) = 0$. For the second derivatives, we know that they must have the general form of an isotropic rank-4 tensor [7],

$$Q^{ik}_{jn} = -\partial_j\partial_n R_{ik}(\mathbf{0}) = A\delta_{ik}\delta_{jn} + B\delta_{ij}\delta_{kn} + C\delta_{in}\delta_{jk}. \tag{S20}$$

By definition, this tensor must be symmetric under exchange of j and n, which implies $B = C$. Finally, incompressibility implies $\delta_{ij}Q^{ik}_{jn} = 0$ so that

$$A = -4B, \tag{S21}$$

leading to the general form [8]

$$Q^{ik}_{jn} = Q(4\delta_{ik}\delta_{jn} - \delta_{ij}\delta_{kn} - \delta_{in}\delta_{jk}). \tag{S22}$$

Analogous arguments can be used to deduce the general form (59) of the fourth-order derivatives of the correlation tensor. Using this expression, our term M can be evaluated,

$$\begin{aligned} M &= -\frac{1}{2}Q\partial_\kappa\left((4\delta_{ik}\delta_{jn} - \delta_{ij}\delta_{kn} - \delta_{in}\delta_{jk})\kappa\left\langle|\partial_\phi\mathbf{L}|\hat{n}_i\hat{n}_j\delta(\kappa-\tilde{\kappa})(\hat{n}_k\hat{n}_n-2\hat{t}_k\hat{t}_n)\right\rangle\right) \\ &= -2Q\partial_\kappa\left(\kappa f(\kappa;t)\right)\left\langle|\partial_\phi\mathbf{L}|\right\rangle, \end{aligned} \tag{S23}$$

by orthonormality of the Frenet-Serret frame.

In the following, we list all the terms that need to be computed along with the results of their evaluation. By Gaussian integration by parts and the product rule for functional derivatives, the averages of (S14) split into

$$\langle|\partial_\phi\mathbf{L}|\delta(\kappa-\tilde{\kappa})\hat{t}_i\hat{t}_j\partial_j u_i\rangle = \int \mathrm{d}^3\mathbf{z}\;\left\langle\left(\partial_j R_{ik}(\mathbf{L}(\phi,t)-\mathbf{z})\right)\delta(\kappa-\tilde{\kappa})\hat{t}_i\hat{t}_j\frac{\delta|\partial_\phi\mathbf{L}|}{\delta u_k(\mathbf{z},t)}\right\rangle \tag{S24}$$

$$+\int \mathrm{d}^3\mathbf{z}\;\left\langle\left(\partial_j R_{ik}(\mathbf{L}(\phi,t)-\mathbf{z})\right)|\partial_\phi\mathbf{L}|\hat{t}_i\hat{t}_j\frac{\delta\left[\delta(\kappa-\tilde{\kappa})\right]}{\delta u_k(\mathbf{z},t)}\right\rangle \tag{S25}$$

$$+\int \mathrm{d}^3\mathbf{z}\;\left\langle\left(\partial_j R_{ik}(\mathbf{L}(\phi,t)-\mathbf{z})\right)|\partial_\phi\mathbf{L}|\delta(\kappa-\tilde{\kappa})\hat{t}_j\frac{\delta\hat{t}_i}{\delta u_k(\mathbf{z},t)}\right\rangle \tag{S26}$$

$$+\int \mathrm{d}^3\mathbf{z}\;\left\langle\left(\partial_j R_{ik}(\mathbf{L}(\phi,t)-\mathbf{z})\right)|\partial_\phi\mathbf{L}|\delta(\kappa-\tilde{\kappa})\hat{t}_i\frac{\delta\hat{t}_j}{\delta u_k(\mathbf{z},t)}\right\rangle \tag{S27}$$

$$+\int \mathrm{d}^3\mathbf{x}\int \mathrm{d}^3\mathbf{z}\;\left(\partial_j R_{ik}(\mathbf{x}-\mathbf{z})\right)\left\langle|\partial_\phi\mathbf{L}|\delta(\kappa-\tilde{\kappa})\hat{t}_i\hat{t}_j\frac{\delta\left[\delta(\mathbf{x}-\mathbf{L}(\phi,t))\right]}{\delta u_k(\mathbf{z},t)}\right\rangle, \tag{S28}$$

$$\langle|\partial_\phi\mathbf{L}|\hat{t}_i\hat{t}_j\partial_j u_i\rangle = \int \mathrm{d}^3\mathbf{z}\;\left\langle\left(\partial_j R_{ik}(\mathbf{L}(\phi,t)-\mathbf{z})\right)\hat{t}_i\hat{t}_j\frac{\delta|\partial_\phi\mathbf{L}|}{\delta u_k(\mathbf{z},t)}\right\rangle \tag{S29}$$

$$+\int \mathrm{d}^3\mathbf{z}\;\left\langle\left(\partial_j R_{ik}(\mathbf{L}(\phi,t)-\mathbf{z})\right)|\partial_\phi\mathbf{L}|\hat{t}_j\frac{\delta\hat{t}_i}{\delta u_k(\mathbf{z},t)}\right\rangle \tag{S30}$$

$$+\int \mathrm{d}^3\mathbf{z}\;\left\langle\left(\partial_j R_{ik}(\mathbf{L}(\phi,t)-\mathbf{z})\right)|\partial_\phi\mathbf{L}|\hat{t}_i\frac{\delta\hat{t}_j}{\delta u_k(\mathbf{z},t)}\right\rangle \tag{S31}$$

$$+\int \mathrm{d}^3\mathbf{x}\int \mathrm{d}^3\mathbf{z}\;\left(\partial_j R_{ik}(\mathbf{x}-\mathbf{z})\right)\left\langle|\partial_\phi\mathbf{L}|\hat{t}_i\hat{t}_j\frac{\delta\left[\delta(\mathbf{x}-\mathbf{L}(\phi,t))\right]}{\delta u_k(\mathbf{z},t)}\right\rangle, \tag{S32}$$

$$\langle |\partial_\phi \mathbf{L}| \delta(\kappa-\tilde\kappa) \hat n_i \hat n_j \partial_j u_i\rangle = \int \mathrm{d}^3\mathbf{z}\ \left\langle \big(\partial_j R_{ik}(\mathbf{L}(\phi,t)-\mathbf{z})\big)\, \delta(\kappa-\tilde\kappa)\hat n_i \hat n_j \frac{\delta |\partial_\phi \mathbf{L}|}{\delta u_k(\mathbf{z},t)}\right\rangle \tag{S33}$$

$$+ \int \mathrm{d}^3\mathbf{z}\ \left\langle \big(\partial_j R_{ik}(\mathbf{L}(\phi,t)-\mathbf{z})\big)\, |\partial_\phi \mathbf{L}| \hat n_i \hat n_j \frac{\delta\left[\delta(\kappa-\tilde\kappa)\right]}{\delta u_k(\mathbf{z},t)}\right\rangle \tag{S34}$$

$$+ \int \mathrm{d}^3\mathbf{z}\ \left\langle \big(\partial_j R_{ik}(\mathbf{L}(\phi,t)-\mathbf{z})\big)\, |\partial_\phi \mathbf{L}| \delta(\kappa-\tilde\kappa) \hat n_j \frac{\delta \hat n_i}{\delta u_k(\mathbf{z},t)}\right\rangle \tag{S35}$$

$$+ \int \mathrm{d}^3\mathbf{z}\ \left\langle \big(\partial_j R_{ik}(\mathbf{L}(\phi,t)-\mathbf{z})\big)\, |\partial_\phi \mathbf{L}| \delta(\kappa-\tilde\kappa) \hat n_i \frac{\delta \hat n_j}{\delta u_k(\mathbf{z},t)}\right\rangle \tag{S36}$$

$$+ \int \mathrm{d}^3\mathbf{x} \int \mathrm{d}^3\mathbf{z}\ \big(\partial_j R_{ik}(\mathbf{x}-\mathbf{z})\big) \left\langle |\partial_\phi \mathbf{L}| \delta(\kappa-\tilde\kappa) \hat n_i \hat n_j \frac{\delta\left[\delta(\mathbf{x}-\mathbf{L}(\phi,t))\right]}{\delta u_k(\mathbf{z},t)}\right\rangle, \tag{S37}$$

and

$$\langle |\partial_\phi \mathbf{L}| \delta(\kappa-\tilde\kappa) \hat n_i \hat t_j \hat t_k \partial_j \partial_k u_i\rangle = \int \mathrm{d}^3\mathbf{z}\ \left\langle \big(\partial_j \partial_k R_{il}(\mathbf{L}(\phi,t)-\mathbf{z})\big)\, \delta(\kappa-\tilde\kappa)\hat n_i \hat t_j \hat t_k \frac{\delta |\partial_\phi \mathbf{L}|}{\delta u_l(\mathbf{z},t)}\right\rangle \tag{S38}$$

$$+ \int \mathrm{d}^3\mathbf{z}\ \left\langle \big(\partial_j \partial_k R_{il}(\mathbf{L}(\phi,t)-\mathbf{z})\big)\, |\partial_\phi \mathbf{L}| \hat n_i \hat t_j \hat t_k \frac{\delta\left[\delta(\kappa-\tilde\kappa)\right]}{\delta u_l(\mathbf{z},t)}\right\rangle \tag{S39}$$

$$+ \int \mathrm{d}^3\mathbf{z}\ \left\langle \big(\partial_j \partial_k R_{il}(\mathbf{L}(\phi,t)-\mathbf{z})\big)\, |\partial_\phi \mathbf{L}| \delta(\kappa-\tilde\kappa) \hat t_j \hat t_k \frac{\delta \hat n_i}{\delta u_l(\mathbf{z},t)}\right\rangle \tag{S40}$$

$$+ \int \mathrm{d}^3\mathbf{z}\ \left\langle \big(\partial_j \partial_k R_{il}(\mathbf{L}(\phi,t)-\mathbf{z})\big)\, |\partial_\phi \mathbf{L}| \delta(\kappa-\tilde\kappa) \hat n_i \hat t_k \frac{\delta \hat t_j}{\delta u_l(\mathbf{z},t)}\right\rangle \tag{S41}$$

$$+ \int \mathrm{d}^3\mathbf{z}\ \left\langle \big(\partial_j \partial_k R_{il}(\mathbf{L}(\phi,t)-\mathbf{z})\big)\, |\partial_\phi \mathbf{L}| \delta(\kappa-\tilde\kappa) \hat n_i \hat t_j \frac{\delta \hat t_k}{\delta u_l(\mathbf{z},t)}\right\rangle \tag{S42}$$

$$+ \int \mathrm{d}^3\mathbf{x} \int \mathrm{d}^3\mathbf{z}\ \big(\partial_j \partial_k R_{il}(\mathbf{x}-\mathbf{z})\big) \left\langle |\partial_\phi \mathbf{L}| \delta(\kappa-\tilde\kappa) \hat n_i \hat t_j \hat t_k \frac{\delta\left[\delta(\mathbf{x}-\mathbf{L}(\phi,t))\right]}{\delta u_l(\mathbf{z},t)}\right\rangle. \tag{S43}$$

Evaluating each of these terms as explained previously yields

$$
\begin{aligned}
&(\mathrm{S24}) = Qf(\kappa;t)\left\langle|\partial_\phi \mathbf{L}|\right\rangle && (\mathrm{S25}) = \frac{5}{2}Q\partial_\kappa(\kappa f(\kappa;t))\left\langle|\partial_\phi \mathbf{L}|\right\rangle\\
&(\mathrm{S26}) = 4Qf(\kappa;t)\left\langle|\partial_\phi \mathbf{L}|\right\rangle && (\mathrm{S27}) = -Qf(\kappa;t)\left\langle|\partial_\phi \mathbf{L}|\right\rangle\\
&(\mathrm{S28}) = 0 && (\mathrm{S29}) = Q\left\langle|\partial_\phi \mathbf{L}|\right\rangle\\
&(\mathrm{S30}) = 4Q\left\langle|\partial_\phi \mathbf{L}|\right\rangle && (\mathrm{S31}) = -Q\left\langle|\partial_\phi \mathbf{L}|\right\rangle\\
&(\mathrm{S32}) = 0 && (\mathrm{S33}) = -\frac{1}{2}Qf(\kappa;t)\left\langle|\partial_\phi \mathbf{L}|\right\rangle\\
&(\mathrm{S34}) = -2Q\partial_\kappa(\kappa f(\kappa;t))\left\langle|\partial_\phi \mathbf{L}|\right\rangle && (\mathrm{S35}) = \frac{5}{2}Qf(\kappa;t)\left\langle|\partial_\phi \mathbf{L}|\right\rangle\\
&(\mathrm{S36}) = -\frac{5}{2}Qf(\kappa;t)\left\langle|\partial_\phi \mathbf{L}|\right\rangle && (\mathrm{S37}) = 0\\
&(\mathrm{S38}) = 0 && (\mathrm{S39}) = -9P\partial_\kappa f(\kappa;t)\left\langle|\partial_\phi \mathbf{L}|\right\rangle\\
&(\mathrm{S40}) = \frac{9}{\kappa}Pf(\kappa;t)\left\langle|\partial_\phi \mathbf{L}|\right\rangle && (\mathrm{S41}) = 0\\
&(\mathrm{S42}) = 0 && (\mathrm{S43}) = 0,
\end{aligned}
$$

where Q and P are defined through (58) and (59), respectively. Inserting these results into (S14) yields the Fokker-Planck equation (20).

SUPPLEMENTARY NOTE 7. NUMERICAL RESULTS IN THE KRAICHNAN MODEL

Here, we present a numerical analysis of curvature statistics in the Kraichnan model. To this end, we interpret the tracer equation (1) as a Langevin equation. Since Itô and Stratonovich interpretations coincide for this equation, we may use the Euler-Maruyama scheme [9] to integrate particle trajectories. In every time step, the Gaussian flow field is computed on 1024^3 grid points with a model energy spectrum as described by Pope [10, p. 232],

$$E(k) \propto k^{-5/3} f_L(kL) f_\eta(k\eta), \tag{S44}$$

with η a viscous length scale, $L \approx 946\eta$ an integral length scale and the functions

$$f_L(x) = \left(\frac{x}{(x^2 + c_L)^{1/2}} \right)^{11/3} \tag{S45}$$

and

$$f_\eta(x) = \exp\left(-\beta \left(x^4 + c_\eta^4 \right)^{1/4} - c_\eta \right), \tag{S46}$$

which determine the large- and small-scale behavior. The spectrum (S44) integrates to the total energy $E = 1.05$ (code units). The temporal resolution is $\Delta t = 7.15 \times 10^{-7}$ (code units) and the spatial resolution can be quantified by $k_{\max}\eta \approx 2.0$, where $k_{\max}$ is the maximum resolved wavenumber. Furthermore, we choose $\beta = 5.2$, $c_L = 6.03$ and $c_\eta = 0.40$. For particle time stepping, the field is interpolated using spline interpolation with continuous derivatives up to and including third order computed over a kernel of 12^3 grid points. The loops are adaptively refined as described for the Navier-Stokes simulations in Methods.

Figure S11 shows a visualization of an initially circular loop deformed by the Kraichnan field for $1.2Q^{-1}$. Visually, it shares many features of material loops in Navier-Stokes turbulence but appears slightly more compact (compare Fig. 1). The geometric similarities also manifest in the curvature statistics (Figure S12a), which display the same type of unimodal distribution with power-law tails. Over time, the PDF converges to the stationary solution (21) of the Fokker-Planck equation, featuring the power-law tails κ^1 and $\kappa^{-18/7}$.

The Kraichnan model also forms curvature peaks, whose distribution (Figure S12b) qualitatively resembles the one in Navier-Stokes turbulence. Our theory predicts the high-curvature tail to scale as a power law with exponent $-11/7$, which is confirmed by the simulation. In order to form our theory, we made the empirical observation that the curvature peak number grows proportional to the mean line length. This is also what we observe in the Kraichnan model (Figure S13), where the mean line length can be computed analytically to be proportional to e^{4Qt} [11].

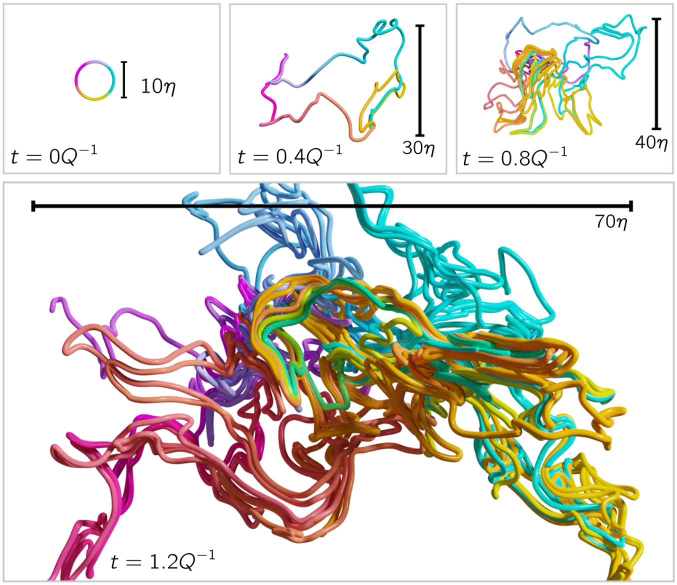

Supplementary Figure S11. **Visualization of a material loop advected by a Kraichnan field** for $1.2Q^{-1}$.

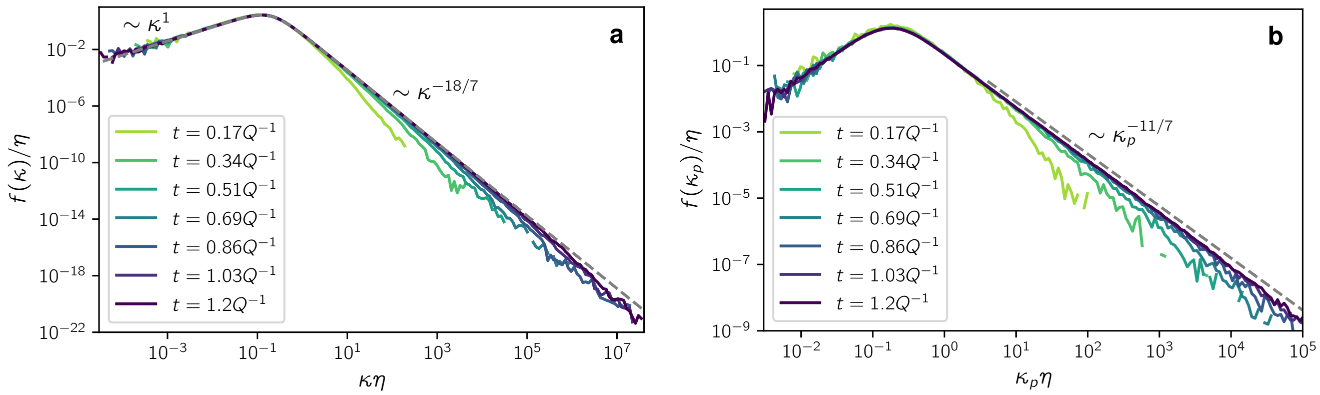

Supplementary Figure S12. **Curvature statistics in the Kraichnan model.** **a** Curvature PDF of material loops at different times. The PDF converges to the stationary solution (21) of the Fokker-Planck equation, indicated by the dashed line. **b** PDF of curvature maxima of material loops at the same times. The high-curvature tail scales as a power law, in agreement with our theoretical prediction $\kappa_p^{-11/7}$.

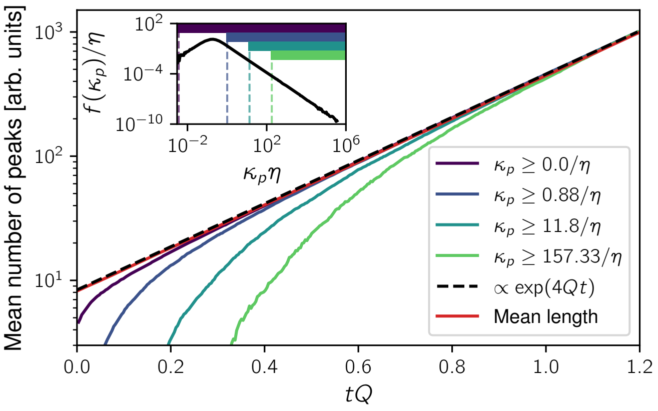

Supplementary Figure S13. **Mean number of curvature peaks in the Kraichnan model** above different thresholds over time, vertically shifted for comparison. Consistent with our observation in Navier-Stokes turbulence, the curves become asymptotically proportional to the mean arc length of loops, which grows as e^{4Qt} as predicted by theory. Inset: Curvature peak distribution at $t = 1.2Q^{-1}$ indicating the different thresholds.

SUPPLEMENTARY NOTE 8. LYAPUNOV EXPONENTS BY QR-DECOMPOSITION

In Methods, we have defined finite-time Lyapunov exponents as the growth rate of singular values of the deformation tensor (32). In practice, however, we instead compute the QR-decomposition of the deformation tensor for numerical stability,

$$F(t) = Q(t)R(t), \tag{S47}$$

where $Q(t)$ is orthogonal and $R(t)$ is upper triangular. Note that the matrix $Q(t)$ is unrelated to the constant Q introduced previously, which quantifies velocity gradient fluctuations in the Kraichnan model. The growth rate of the diagonal elements of $R(t)$ can then be interpreted as an alternative definition of FTLEs [1],

$$\rho_i'(t) = \frac{1}{t}\log R_{ii}(t). \tag{S48}$$

Note that this definition of FTLEs depends on the choice of the coordinate system. Complementing the Methods section, we here show that peak curvature dynamics of a parabola are exactly captured by this alternative definition of FTLEs.

We start from a parabolic material line as defined in (25). Given that the flow is statistically isotropic, FTLE statistics should not depend on the choice of the coordinate system. We can therefore assume that the line is aligned with the coordinate axes, i.e. $\mathbf{k}(0) = \mathbf{e}_1$ and $\mathbf{l}(0) = \mathbf{e}_2$. In this case we have

$$\begin{aligned} \left|\mathbf{k}(t)\right|^2 &= \left|Q(t)R(t)\mathbf{k}(0)\right|^2 & \text{(S49)}\\ &= Q_{ij}(t)R_{j1}(t)Q_{ik}(t)R_{k1}(t) & \text{(S50)}\\ &= R_{11}^2(t), & \text{(S51)} \end{aligned}$$

where we have used the fact that Q is orthogonal and R is upper triangular. Furthermore, we get

$$\begin{aligned} \left|\mathbf{k}(t)\right|^2\left|\mathbf{l}(t)\right|^2 - (\mathbf{k}(t)\cdot\mathbf{l}(t))^2 &= R_{11}^2 Q_{ij}R_{j2}Q_{ik}R_{k2} - (Q_{ij}R_{j1}Q_{ik}R_{k2})^2 & \text{(S52)}\\ &= R_{11}^2(R_{j2}R_{j2}) - (R_{j1}R_{j2})^2 & \\ &= R_{11}^2R_{12}^2 + R_{11}^2R_{22}^2 - R_{11}^2R_{12}^2 & \text{(S53)}\\ &= R_{11}^2(t)R_{22}^2(t). & \text{(S54)} \end{aligned}$$

Hence, the peak curvature given by (30) can be written as

$$\kappa_p(t) = \frac{R_{11}(t)}{R_{22}^2(t)}\kappa_p(0). \tag{S55}$$

Inserting the alternative definition of FTLEs (S48) yields

$$\kappa_p(t) = e^{[\rho_1'(t) - 2\rho_2'(t)]t}\kappa_p(0), \tag{S56}$$

an exact analog to the approximate equation (37). This means that the FTLEs defined by the QR-decomposition precisely capture the curvature growth of parabolic line elements. In that sense, the QR-definition of FTLEs is very suitable for our purposes.

SUPPLEMENTARY REFERENCES

[1] P. L. Johnson and C. Meneveau, Phys. Fluids **27**, 085110 (2015).
[2] C. Bär, *Elementary Differential Geometry* (Cambridge University Press, 2010).
[3] I. T. Drummond and W. Münch, J. Fluid Mech. **225**, 529 (1991).
[4] K. Furutsu, *On the Statistical Theory of Electromagnetic Waves in a Fluctuating Medium (II)*, NBS Monograph No. 79 (U.S. Natl. Inst. Stand., 1964).
[5] M. D. Donsker, Matematika **11**, 128 (1967).
[6] E. A. Novikov, Sov. J. Exp. Theor. Phys. **20**, 1290 (1965).
[7] E. A. Kearsley and J. T. Fong, J. Res. Natl. Inst. Stand. Technol. **79B**, 49 (1975).
[8] A. Pumir, Phys. Rev. Fluids **2**, 074602 (2017).
[9] P. Glasserman, *Monte Carlo Methods in Financial Engineering* (Springer, 2003).
[10] S. B. Pope, *Turbulent Flows* (Cambridge University Press, 2000).
[11] E. Balkovsky and A. Fouxon, Phys. Rev. E **60**, 4164 (1999).
