# Supplementary material for: The statistical geometry of material loops in turbulence
Source: Nat Commun. 2022 Apr 19;13:2088. doi: 10.1038/s41467-022-29422-1 (PMC9018957; doi:10.1038/s41467-022-29422-1)
Supplement: Supplementary file 2 — Description of additional Supplementary File [file 41467_2022_29422_MOESM2_ESM.pdf]

<u>Descriptions of additional supplementary Information Files</u>

Supplementary Movie: Evolution of a material loop advected by a turbulent flow for 27 Kolmogorov times.
